# Preventable Cancer Burden Associated With Poor Diet in the United States

**DOI:** 10.1093/jncics/pkz034

**Published:** 2019-05-22

**Authors:** Fang Fang Zhang, Frederick Cudhea, Zhilei Shan, Dominique S Michaud, Fumiaki Imamura, Heesun Eom, Mengyuan Ruan, Colin D Rehm, Junxiu Liu, Mengxi Du, David Kim, Lauren Lizewski, Parke Wilde, Dariush Mozaffarian

**Affiliations:** 1 Friedman School of Nutrition Science and Policy; 2 School of Medicine, Tufts University, Boston, MA; 3 T. H. Chan School of Public Health, Harvard University, Boston, MA; 4 MRC Epidemiology Unit, University of Cambridge, Cambridge, UK; 5 Department of Epidemiology and Population Health, Albert Einstein College of Medicine, Bronx, NY; 6 Institute for Clinical Research and Health Policy Studies, Tufts Medical Center, Boston, MA

## Abstract

**Background:**

Diet is an important risk factor for cancer that is amenable to intervention. Estimating the cancer burden associated with diet informs evidence-based priorities for nutrition policies to reduce cancer burden in the United States.

**Methods:**

Using a comparative risk assessment model that incorporated nationally representative data on dietary intake, national cancer incidence, and estimated associations of diet with cancer risk from meta-analyses of prospective cohort studies, we estimated the annual number and proportion of new cancer cases attributable to suboptimal intakes of seven dietary factors among US adults ages 20 years or older, and by population subgroups.

**Results:**

An estimated 80 110 (95% uncertainty interval [UI] = 76 316 to 83 657) new cancer cases were attributable to suboptimal diet, accounting for 5.2% (95% UI = 5.0% to 5.5%) of all new cancer cases in 2015. Of these, 67 488 (95% UI = 63 583 to 70 978) and 4.4% (95% UI = 4.2% to 4.6%) were attributable to direct associations and 12 589 (95% UI = 12 156 to 13 038) and 0.82% (95% UI = 0.79% to 0.85%) to obesity-mediated associations. By cancer type, colorectal cancer had the highest number and proportion of diet-related cases (n = 52 225, 38.3%). By diet, low consumption of whole grains (n = 27 763, 1.8%) and dairy products (n = 17 692, 1.2%) and high intake of processed meats (n = 14 524, 1.0%) contributed to the highest burden. Men, middle-aged (45–64 years) and racial/ethnic minorities (non-Hispanic blacks, Hispanics, and others) had the highest proportion of diet-associated cancer burden than other age, sex, and race/ethnicity groups.

**Conclusions:**

More than 80 000 new cancer cases are estimated to be associated with suboptimal diet among US adults in 2015, with middle-aged men and racial/ethnic minorities experiencing the largest proportion of diet-associated cancer burden in the United States.

Cancer is the second leading cause of death in the United States, accounting for 1 in 4 deaths (1). In 2018, an estimated 1.7 million Americans were newly diagnosed with cancer, and 0.6 million will die from cancer (1). The associated economic burden in the United States exceeds $80 billion annually for direct medical costs alone (2,3). With population aging, escalating health-care costs, and increasing rates of risk factors, such as obesity, the cancer burden is projected to further increase (4,5).

Poor dietary habits have long been recognized to be associated with cancer risk (6,7). With the recent dietary data (8) and cancer incidence (9) in the United States, and updated evidence on nutrition and cancer risk (10), the cancer burden associated with various dietary factors needs to be evaluated. Importantly, obesity has been recognized as an important risk factor for 13 cancers (11). The diet-associated cancer burden mediated through obesity has not yet been formally quantified. In addition, disparities in diet-associated cancer burden, such as by age, sex, and race/ethnicity, are not well established. To address these questions, we estimated the preventable cancer burden associated with suboptimal intake of seven dietary factors, individually and combined, among US adults for 15 cancers. We separately estimated the cancer burden attributable to direct associations with poor diet and that attributable to obesity-mediated associations. Accounting for demographic differences in dietary intake and cancer incidence, we further estimated the diet-associated cancer burden among age, sex, and race/ethnicity subgroups.

## Methods

### Study Design

We used a population-based comparative risk assessment (CRA) model to estimate the number of cancer cases associated with suboptimal diet among US adults (Supplementary Appendix 1, available online). The model incorporated data and corresponding uncertainty on 1) dietary intake among US adults by age, sex, and race/ethnicity; 2) relative risk estimates for diet and cancer risk; 3) relative risk estimates for body mass index (BMI) and cancer risk; 4) effect estimates of changes in diet with change in BMI; 5) optimal distribution of these dietary factors; and 6) cancer incidence by age, sex, and race/ethnicity (Table 1). The study is exempt for ethical review and waived for consent.

**Table 1.** Dietary factors, current intake in US adults aged 20 years or older in 2011–2014, optimal intake, related cancer outcomes, cancer relative risks, and effect estimates on body mass index

Dietary factor	Current intake		Optimal intake	Mean (SD), g/d	Cancer outcome	Unit of RR	Diet–cancer RR (95% CI)†	Effect estimates on diet–BMI kg/m^2^ (95% CI), per 1 serving/d‡	
	Mean (SD)	Median (IQR)*	Mean (SD)					Baseline BMI <25	Baseline BMI ≥25
Fruits, servings/d§	0.72 (0.55)	0.58 (0.72)	3 (0.3) servings/d	300 (30)	Mouth, pharynx, and larynx	1 serving/d (100 g/d)	0.95 (0.91 to 1.00)	−0.06 (−0.08 to −0.04)	−0.11 (−0.16 to −0.06)
Vegetables, servings/d§	1.16 (0.43)	1.12 (0.54)	4 (0.4) servings/d	400 (40)	Mouth, pharynx, and larynx	1 serving/d (100 g/d)	0.91 (0.87 to 0.96)	−0.03 (−0.04 to −0.01)	−0.06 (−0.09 to −0.02)
Whole grains, servings/d	0.93 (0.59)	0.80 (0.88)	—	125 (12.5)	Colon and rectum	90 g/d	0.83 (0.78 to 0.89)	−0.05 (−0.07 to −0.03)	−0.08 (−0.10 to −0.06)
Processed meats, servings/d	0.87 (0.39)	0.78 (0.53)	No intake	—	Colon and rectum, stomach cancer (noncardia)	1 serving/d(50 g/d)	1.16 (1.08 to 1.26) 1.18 (1.01 to 1.38)	0.13 (0.07 to 0.19)	0.16 (0.11 to 0.21)
Red meats, servings/d	1.47 (0.43)	1.40 (0.57)	1 (0.1) serving/wk	14.3 (1.4)	Colon and rectum	1 serving/d (100 g/d)	1.12 (1.00 to 1.25)	0.13 (0.07 to 0.20)	0.23 (0.14 to 0.32)
Total dairy products, servings/d	1.40 (0.43)	1.38 (0.59)	3 (0.3) servings /d	—	Colon and rectum	1.6 serving/d (400 g/d)	0.87 (0.83 to 0.90)	No effect estimates of total dairy products on BMI	
SSBs, servings/d	1.08 (0.55)	0.63 (1.27)	No intake	—	13 cancers through obesity	No direct RR of SSB on cancer		0.09 (0.05 to 0.14)	0.23 (0.14 to 0.32)

* Means, SDs, medians, IQRs, and percent were estimated using the National Cancer Institute method and adjusted for National Health and Nutrition Examination Survey dietary weights to account for the complex survey design (including oversampling), survey nonresponse, and poststratification. BMI = body mass index, CI = confidence interval; IQR = interquartile range; RR = relative risk; SSB = sugar-sweetened beverages.

† RR estimates were based on meta-analyses of prospective cohort studies with limited evidence of bias from confounding, where the associations were multivariable adjusted and independent of obesity (Supplementary Table 1, available online).

‡ Obesity is associated with an increased risk of 13 cancers (Supplementary Table 2, available online). Although there is no direct RR for SSB and cancer, SSB can increase the risk of cancer mediated through obesity.

§ Fruits exclude fruit juices, and vegetables exclude starchy vegetables.

### Current and Optimal Distribution of Dietary Intake

Current distribution of dietary intake was estimated using a nationally representative sample of US adults who participated in the two most recent cycles of the National Health and Nutrition Examination Survey (NHANES) (2013–2014 and 2015–2016) (12). Complex survey design and sampling weights were accounted to represent the dietary intake of the US population ages 20 years or older, and in population subgroups. To correct for the measurement error, we applied the National Cancer Institute (NCI) method to estimate usual intake and distribution for all seven dietary factors (Supplementary Appendix 1, available online) (13). As documented in prior literature, the NCI method is the preferred method for estimating usual intake distribution from 24-hour diet recalls (14). Optimal distribution was characterized based on the intake associated with lowest disease risk, assessed by the Global Burden of Disease (GBD) 2010 (15).

### Selection of Dietary Factors

The World Cancer Research Fund and the American Institute for Cancer Research (WCRF/AICR) have performed systematic reviews to evaluate the evidence of various dietary factors on cancer incidence and mortality (10). For each diet-cancer relationship, the strength of evidence was categorized into “convincing,” “probable,” “limited-suggestive,” “limited-no conclusion,” and “substantial effect unlikely.” We selected dietary factors having “convincing” or “probable” evidence on cancer risk: fruits, vegetables, whole grains, processed meats, red meats, and total dairy products. Sugar-sweetened beverage (SSB) was not assessed as a separate food group in WCRF/AICR reports, but its causal impact on adiposity provides strong support to include SSB as a dietary factor for cancer prevention (16–18).

### Etiologic Relationships between Diet and Cancer

Methods for reviewing and synthesizing evidence to estimate relative risks (RRs) for direct diet-cancer associations are described in Supplementary Appendices 2 and 3 (available online). The present analysis incorporated the relative risk estimates from meta-analyses of prospective cohort studies with limited evidence of bias from confounding, where the associations were multivariable adjusted and independent of BMI (Supplementary Table 1, available online). To separately estimate diet-related cancer burden that is mediated by obesity, we connected the effect of changes in dietary factors (eg, SSB) on change in BMI (the diet-BMI effect size) to the association of BMI with cancer risk (the BMI-cancer RR) (Supplementary Appendix 1, available online). The diet-BMI effect size was estimated based on pooled analysis from 120 977 US men and women in three prospective cohort studies (Supplementary Table 3, available online, 19). The BMI-cancer relative risk for 13 cancers was based on the meta-analysis conducted by the International Agency for Research on Cancer (IARC) (11) and WCRF/AICR Continuous Update Project reports (10) (Supplementary Table 2, available online).

### Incident Cancer Cases by Age, Sex, and Race/Ethnicity

The 2015 cancer incidence was obtained from the Centers for Disease Control and Prevention’s National Program for Cancer Registries and the NCI’s Surveillance, Epidemiology, and End Results program, which collectively provided a complete enumeration of cancer cases for the US population (20). Cases for individual cancer types were obtained by applying the International Classification for Diseases for Oncology third edition codes corresponding to primary cancer site. Additional specifications on tumor histologic types and anatomic locations were used to obtain the cancer cases for esophageal adenocarcinoma and stomach cardia and noncardia cancers (Supplementary Appendix 4, available online).

### Statistical Analysis

We adapted the GBD CRA framework (21,22) that estimates the population-attributable fraction (PAF) (23), which estimates the cancer burden attributable to suboptimal diet by comparing the current distribution of dietary intake patterns to the distribution of optimal intake in each age, sex, and race/ethnicity stratum. The joint PAF of seven dietary factors was estimated by proportional multiplication of each stratum-specific PAF using the conventional Mant and Hicks formula (24) for cumulative effects.

Uncertainty was quantified using multiway probabilistic Monte Carlo simulations, jointly incorporating stratum-specific uncertainties in dietary intake, cancer incidence, diet-cancer relative risks, BMI-cancer relative risks, and diet-BMI effect sizes. Corresponding 95% uncertainty intervals (UIs) were derived from the 2.5th and 97.5th percentiles of 1000 estimates. All analyses were performed using R statistical software, version 3.4.1. (R Foundation for Statistical Computing, Vienna, Austria).

## Results

In 2015, an estimated 80 110 (95% UI = 76 316 to 83 657) new cancer cases were associated with suboptimal intake of seven dietary factors including low intake of vegetables, fruits, and whole grains and high intake of processed meats, red meats, total dairy products, and SSB, accounting for 5.2% (95% UI = 5.0% to 5.5%) of all invasive cancers among US adults ages 20 years or older (Table 2).

**Table 2.** Annual cancer cases and population- attributable fraction for suboptimal dietary intake among US adults aged 20 years or older in 2015, by cancer type

Cancer burden by cancer type	New cancer cases No. (95% UI)*	Population- attributable fraction % (95 UI)†
Colon and rectum	52 225 (49 263 to 55 302)	38.3 (36.1 to 40.4)
Mouth, pharynx, and larynx	14 421 (12 492 to 16 146)	25.9 (22.6 to 28.9)
Corpus uteri	3165 (2590 to 3406)	6.08 (5.67 to 6.53)
Breast (postmenopausal)	3059 (2786 to 3335)	1.57 (1.43 to 1.71)
Kidney	2017 (1907 to 2132)	3.37 (3.19 to 3.55)
Stomach	1564 (1179 to 1922)	6.82 (5.20 to 8.43)
Liver	1000 (924 to 1080)	3.29 (3.06 to 3.58)
Pancreas	538 (491 to 583)	1.19 (1.09 to 1.30)
Esophagus (adenocarcinoma)	475 (431 to 527)	4.62 (4.23 to 5.07)
Thyroid	415 (374 to 460)	0.88 (0.80 to 0.97)
Prostate (advanced)	274 (215 to 335)	0.92 (0.72 to 1.13)
Multiple myeloma	240 (214 to 270)	1.10 (0.98 to 1.23)
Ovary	173 (146 to 199)	0.84 (0.71 to 0.97)
Gallbladder	105 (95 to 117)	2.81 (2.59 to 3.07)
Total	80 110 (76 316 to 83 657)	5.23 (4.98 to 5.46)

* For each cancer type, the total number of cancer incidence attributable to poor diet = the total number of specific cancer incidence × PAF. Cancer incidence that occurred in the US adult population in 2015 were used in the above calculations. PAF = population-attributable fraction; UI = uncertainty Intervals.

† For each cancer type, the PAF was estimated using the joint PAF for all dietary factors included in this analysis (fruits, nonstarchy vegetables, whole grains, processed meats, red meats, total dairy products, and sugar sweetened beverages). Joint PAF = 1 - (1-PAF dietary target_1_) × (1-PAF dietary target_2_) ×…× (1-PAF dietary target_n_). Because of the overlap between the effects of different factors, the joint PAF for all dietary factors combined is less than the sum of the PAFs associated with each dietary target.

**Figure1.**
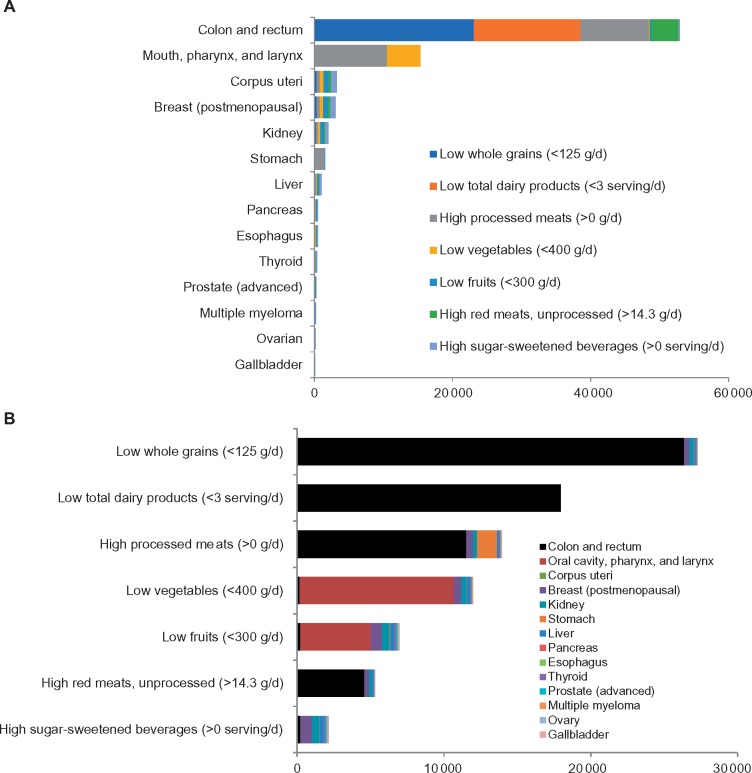
Estimated cancer burden attributable to suboptimal diet among US adults in 2015. **A**) By cancer type. **B**) By dietary factors.

The largest number of cancer cases associated with poor diet was for cancer of the colon and rectum (n = 52 225), followed by cancer of the mouth, pharynx, and larynx (n = 14 421); corpus uteri (n = 3165); breast (postmenopausal) (n = 3059); kidney (n = 2017); stomach (n = 1564); liver (n = 1000); pancreas (n = 538); esophagus (adenocarcinoma) (n = 475); thyroid (n = 415); prostate (advanced) (n = 274); multiple myeloma (n = 240); ovary (n = 173); and gallbladder (n = 105) (Figure 1). The highest proportion (PAF) of cancer cases associated with diet was for colorectal cancer (38.3%), followed by cancer of the mouth, pharynx, and larynx (25.9%); stomach (6.8%); corpus uteri (6.1%); esophagus (adenocarcinoma) (4.6%); kidney (3.9%); liver (3.1%); gallbladder (2.8%); breast (postmenopausal) (1.5%); pancreas (1.2%); multiple myeloma (1.1%); prostate (advanced) (0.9%); thyroid (0.9%); and ovary (0.8%) (Supplementary Figure 1 and Supplementary Table 4, available online).

Insufficient whole grain intake accounted for the largest number and proportion of cancer cases in 2015 (n = 27 763, 1.8%), followed by insufficient dairy intake (n = 17 962, 1.2%); high processed meat intake (n = 14 524, 1.0%); insufficient vegetable intake (n = 12 663, 0.8%); insufficient fruit intake (n = 7927, 0.5%); high red meat intake (n = 5689, 0.4%); and high SSB intake (n = 3119, 0.2%) (Figure 1 and Supplementary Figure 2, available online). Of the diet-associated cancer cases, 67 488 (95% UI = 63 583 to 70 978) and 4.4% (95% UI = 4.2% to 4.6%) were attributed to direct associations with suboptimal diet, and 12 589 (95% UI = 12 156 to 13 038) and 0.82% (95% UI = 0.79% to 0.85%) were attributed to BMI-mediated associations (Table 3). The three leading dietary factors attributable to cancer burden through direct associations were insufficient whole grain intake, insufficient dairy intake, and excess processed meat intake, accounting for 26 268 (1.7%), 17 692 (1.2%), and 12 741 (0.8%) new cancer cases, respectively; and the two leading dietary factors attributable to cancer burden through BMI-mediated associations were low fruit intake and high SSB consumption, accounting for 3129 (0.2%) and 3119 (0.2%) of new cancer cases, respectively.

**Table 3.** Annual cancer cases and population -attributable fraction for suboptimal dietary intake among US adults aged 20 years in 2015, by dietary factor

Cancer burden by dietary factor	Total diet-associated cancer burden		Cancer burden attributable to direct associations*		Cancer burden attributable to BMI-mediated associations*	
	No. of cases (95% UI)†	% PAF (95 UI)†	No. of cases (95% UI)†	% PAF (95 UI)†	No. of cases (95% UI)†	% PAF (95 UI)†
Insufficient whole grains, <3 servings/d	27 763 (24 734 to 30 596)	1.81 (1.61 to 2.00)	26 268 (23 241 to 29 096)	1.72 (1.52 to 1.90)	1494 (1396 to 1600)	0.10 (0.09 to 0.10)
Total dairy products, <3 servings/d	17 962 (16 317 to 19 572)	1.17 (1.07 to 1.28)	17 962 (16 317 to 19 572)	1.17 (1.07 to 1.28)	0	0
High processed meats, >0 serving/d	14 524 (12 473 to 16 752)	0.95 (0.81 to 1.09)	12 741 (10 715 to 14 966)	0.83 (0.70 to 0.98)	1770 (1652 to 1924)	0.12 (0.11 to 0.13)
Insufficient vegetables, <4 servings g/d	12 663 (11 026 to 14 119)	0.83 (0.72 to 0.92)	10 532 (8902 to 12 060)	0.69 (0.58 to 0.79)	2111 (1912 to 2340)	0.14 (0.12 to 0.15)
Insufficient fruits, <3 servings g/d	7927 (6752 to 9146)	0.52 (0.44 to 0.60)	4787 (3632 to 6053)	0.31 (0.24 to 0.39)	3129 (2891 to 3391)	0.20 (0.19 to 0.22)
High red meats, >1 serving/wk	5689 (4168 to 7332)	0.37 (0.27 to 0.48)	4511 (2983 to 6165)	0.29 (0.19 to 0.40)	1185 (1089 to 1289)	0.08 (0.07 to 0.08)
High sugar sweetened beverages, >0 serving/d	3119 (2891 to 3352)	0.20 (0.19 to 0.22)	0	0	3119 (2891 to 3352)	0.20 (0.19 to 0.22)
All dietary targets‡	80 110 (76 316 to 83 657)	5.23 (4.98 to 5.46)	67 488 (63 583 to 70 978)	4.40 (4.15 to 4.64)	12 589 (12 156 to 13 038)	0.82 (0.79 to 0.85)

* Direct cancer burden was estimated based on the direct diet-cancer RRs. Indirect cancer burden was estimated based on BMI-mediated diet-cancer associations by linking diet-BMI estimates and BMI-cancer RRs (Supplementary Appendix 1, available online). BMI = body mass index; PAF = population-attributable fraction; RR = relative risk; UI = uncertainty intervals.

† The total number of cancer incidence attributable to each dietary target was obtained by summing the numbers of each cancer type. The PAF for each dietary target was calculated by dividing the total number of cancer incidence attributable to each dietary factor by the total number of cancer incidences (all sites) that occurred in the US adult population in 2015.

‡ The PAF of all dietary targets was estimated using the joint PAF. Because of the overlap between the effects of different dietary targets, the joint PAF for all dietary factors combined is less than the sum of the PAFs associated with each dietary target. Combined PAF = 1 - (1-PAF dietary target_1_) × (1-PAF dietary target_2_) ×…× (1-PAF dietary target_n_). The total number of cancer incidence attributable to all dietary factors was calculated by the product of the total number of cancer incidence (all sites) × combined PAF.

### Diet-Attributed Cancer Burden by Age, Sex, and Race/Ethnicity

**Figure 2.**
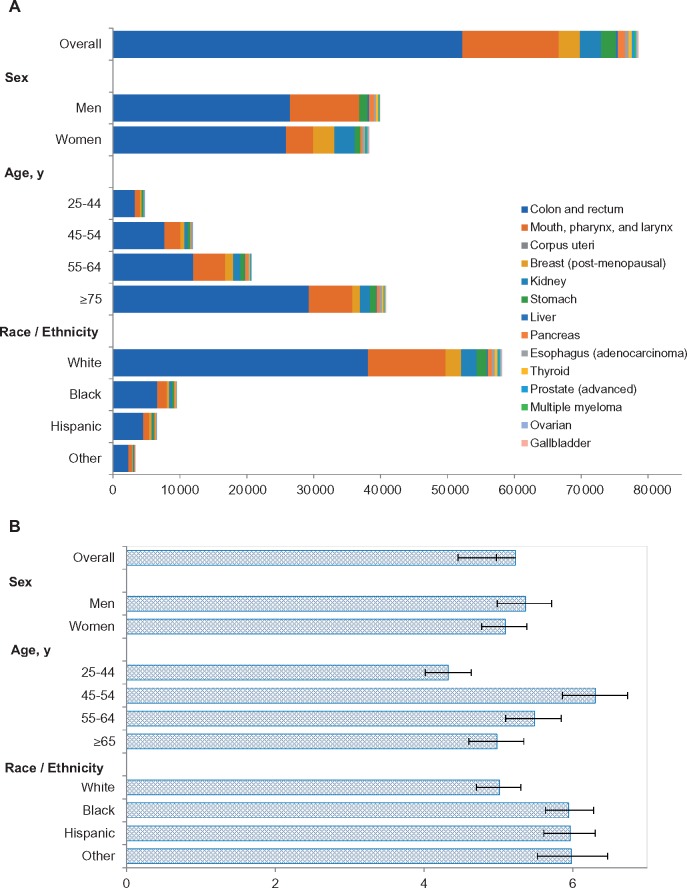
Estimated cancer burden attributable to suboptimal diet among US adults in 2015 among population subgroups. **A**) Number of new cancer cases. **B**) Population- attributable fraction in percentage.

The number of diet-associated cancer cases and PAFs were both higher among men than women (Figure 2 and Supplementary Tables 5 and 6, available online). As expected, the number of diet-associated cancer cases was highest among older adults (age ≥65 years), whereas the middle age groups (45–54 and 55–64 years) overall had higher PAFs than younger or older individuals. Racial/ethnic minorities (non-Hispanic blacks, Hispanics, and others) had higher PAFs for the overall cancer burden than non-Hispanic whites. Across age, sex, and race/ethnicity groups, the top five dietary factors associated with cancer burden in the United States were whole grains, dairy products, processed meats, vegetables, and fruits.

## Discussion

Based on a CRA model and nationally representative data, we estimated that more than 80 000 new cancer cases in 2015 were associated with suboptimal intake of seven dietary factors among US adults. Among the dietary factors evaluated, low consumption of whole grains and high intake of processed meats were associated with the largest number of new cancer cases. Suboptimal diet was associated with the most cases for colorectal cancer among all cancers.

Our results suggest that suboptimal diet was associated with 5.2% of all invasive cancer cases in the United States. Compared to the estimated cancer burden with other modifiable risk factors (25), diet-associated cancer burden was comparable to that with alcohol intake (4%–6%), slightly lower than excessive body weight (7%–8%), and higher than physical inactivity (2%–3%). Although the cancer burden attributable to diet might be smaller than that for lack of screening such as mammography for breast cancer (26) and colonoscopy for colorectal cancer (27), population-based strategies to improve diet could associate with lower cost and represent a cost-effective approach to reduce cancer burden, especially among low-income Americans. These results reinforce the importance of addressing unhealthy diet at the population level and evaluating the cost-effectiveness of broad nutrition policies on reducing cancer burden and disparities in the United States.

Insufficient whole grain consumption and low dairy intake were the two leading dietary factors associated with the preventable cancer burden in the United States. Although whole grain consumption has been modestly improved in the past decade (mean intake increased from 0.6 serving per day in 1999–2000 to 1 serving per day in 2013–2014), it still falls short of the recommended intake (3 servings per day) (28,29). Following the scientific consensus of the health benefits of whole grains, the federal dietary guidelines have explicitly recommended half of the grain consumption to be whole grains. However, less than 20% of the grains consumed by US adults were whole grains (29,30). Lack of public awareness of the health benefits of whole grains and lack of knowledge to identify whole grain products may contribute to their low consumption (31). Our results call for nutrition policies to address US cancer burden by improving Americans’ whole grain consumption, such as standard government-led whole grain labels paired with education. The current level of dairy consumption (1.40 servings per day) among US adults is less than half of the 3 daily servings recommended by the 2015–2020 Dietary Guidelines for Americans (32). Our modeled estimates suggest that increasing dairy consumption to the recommended level would result in a meaningful reduction in colorectal cancer cases among US adults, given the strong evidence for a protective association (33). Although some cancer guidelines do not explicitly recommend an increase in dairy consumption (10), potentially because of the still limited evidence that dairy consumption may increase the risk of prostate cancer (34), dairy products are considered to fit in a cancer-protective diet.

Excessive processed meat consumption is the third leading dietary factor associated with cancer burden among US adults. Unlike the red meat consumption that showed a decreasing trend, the consumption of processed meats remained unchanged in the past 15 years (28,29). US adults consumed, on average, about 1 oz of processed meats daily (29), more than twice the recommended intake by the American Heart Association (ie, <0.5 oz per day). Despite the classification of processed meat as “carcinogenic to human” (group 1) by IARC (35), there is a lack of public awareness of the harms of processed meat consumption on cancer risk (36). Our findings may stimulate the policy discussion for reducing processed meat consumption in the United States, such as including health warning labels on food items that contain processed meats, disincentivizing the use and provision of processed meats in fast-food restaurants, and limiting processed meats from school meal programs and workplace cafeterias.

Obesity has been recognized as an important risk factor for 13 cancers (10,11). By associating long-term change in BMI as a result of change in diet, we estimated that approximately 16% of the 80 110 diet-associated cancer cases were attributable to the obesity-mediated pathways. To the best of our knowledge, this provides the first estimate of the cancer burden attributable to BMI-mediated associations. This proportion may be underestimated because we have not incorporated diet-associated obesity in early life, which strongly predicts obesity in adulthood (37). Given the long induction period of diet on cancer risk and susceptibility of early life dietary exposure (38), cancer prevention strategies focusing on American youth, such as restricting SSB in schools and imposing stronger quality standards to school meals, may play important roles in reducing cancer burden in the United States (39).

Larger numbers of diet-associated cancer cases were estimated in men than in women, which reflected both the worse dietary intake and the higher cancer incidence in men. Middle-age Americans (45–64 years) had a higher proportion of cancer cases attributable to poor diet than younger or older adults. Such an age disparity may reflect a combination of higher cancer incidence in middle-age than younger adults, and worse dietary intake in middle-age than older adults. Suboptimal diet accounted for a higher proportion of cancer burden attributable to diet among non-Hispanic blacks, Hispanics, and others than non-Hispanic whites, largely because of a suboptimal diet in racial/ethnic minorities. Disparities in diet-associated cancer burden should guide public health planning for the at-risk groups.

Diet-associated cancer burden was lower in our study than some early estimates in United States (40) and the United Kingdom (41), ranging from 7% to 10%, but was comparable to the estimate from a recent study in the United States (25): the PAFs for each of the five dietary factors (fruits and vegetables, fiber, processed meat, red meat, and calcium) ranged from 0.5% to 2.2%, and together the five dietary factors contributed to about 4.5% of the total new cancer cases among US adults aged 30 years or older in 2014. We based our analysis on slightly different dietary factors. We included SBSs and estimated cancer burden attributable to BMI-mediated associations. We included whole grains and dairy products but not dietary fiber and calcium. Although focusing on nutrient (eg, fiber or calcium) provides a more complete picture of the PAF for that nutrient, evaluating food (eg, whole grains or dairy products) considers the inherent interactions among nutrients from the same food. For example, the potential mechanisms underlying whole grain consumption and colorectal cancer risk may include not only dietary fiber but also other bioactive compounds such as vitamin E, selenium, lignans, and phenolic compounds (33). Despite these differences, the estimated PAFs were similar: approximately 5% of the cancer burden in the United States is attributable to suboptimal diet. The previous high estimates may reflect stronger relative risk estimates of diet-cancer associations based on case-control studies. For example, pooled estimates from case-control studies estimated that a 50 g/day increment in vegetable intake and a 100 g/day increment in fruit intake was each associated with a 28% reduction in risk of cancer of the mouth, pharynx, and larynx (42). However, our *de novo* meta-analysis using evidence from large-scale prospective cohort studies (43–46) suggested much weaker relative risks: a higher intake of vegetables by 100 g/day was associated with lower risk of the oral cancer types by 9% and of fruit intake, lower risk by 5%. In addition, we did not include fruits and vegetables as dietary factors contributing to lung cancer. It remains controversial whether there is a causal relationship between fruit and vegetable intake and lung cancer. Residual confounding by cigarette smoking is difficult to rule out, and large US and European cohorts reported no associations after accounting for cigarette smoking (47). Given that low consumption of fruits and vegetables is highly prevalent and lung cancer incidence remains high in the United States, the estimated cancer burden attributable to low intake of fruits and vegetables would be greater if lung cancer was included. Notwithstanding these differences, reflecting our best available estimate of relative risks, low intake of fruits and vegetables remains an important dietary target of cancer prevention.

Our study has several strengths. Our model incorporated nationally representative data for the recent dietary intake and cancer incidence among US adults, and the updated diet-cancer risk estimates from the WCRF/AICR reports. In addition to estimating direct diet-cancer associations independent of obesity, our modeling framework incorporated obesity-mediated cancer risks as characterized using published risk estimates for changes in dietary factors and changes in body weight in prospective cohort studies, providing separate estimates of cancer burden attributable to suboptimal diet through obesity-mediated associations. Different from previous studies, we modeled the continuous distribution of dietary factors and used the NCI method to estimate intake distribution, which improves the estimation of usual intake for episodically consumed foods (48). Our model also accounted for the uncertainty of both dietary intake and cancer incidence, allowing estimation of the lower and upper bounds of the plausible effects. Both dietary intake and cancer incidence were modeled with stratum-specific data by age, sex, and race/ethnicity, facilitating estimation of diet-associated cancer disparities.

Potential limitations should also be considered. First, the diet-cancer risk estimates may differ by sex, age, race/ethnicity, and other potential effect modifiers. We used homogeneous relative risk estimates because of the lack of sufficient evidence to support the potentially heterogeneous effects. Second, distribution of diet was estimated based on self-reported dietary intake subject to measurement error. However, the NHANES used interviewer-administered diet recalls, and the two recalls per person were adjusted for energy intake using residual method and averaged whenever possible, each of which reduces measurement error (21). Third, when estimating the cancer burden associated with suboptimal diet, we assumed independence among dietary factors because of the lack of robust estimates of potential interactions among dietary factors. Therefore, the joint estimates for all dietary factors combined may be slightly overestimated. Using a similar approach to estimate joint PAFs, another study assessing diet-associated cardiovascular disease burden suggested that this overestimation is likely to be small (49). By contrast, the large within-person variation in dietary intake is likely to result in underestimation of the etiologic relationships between diet and cancer risk. Fourth, the current estimates have not considered the impact of early life diet on cancer risk, which may further underestimate the cancer burden attributable to suboptimal diet. For example, high consumption of SSB in childhood may result in an increased risk of cancer in adulthood by affecting childhood obesity or growth. Because of the counterfactual nature of the CRA model, the current estimates did not incorporate the induction time of diet affecting cancer risk. The dietary intake patterns were worse 10–15 years ago in the United States (29). Thus, the estimated cancer burden attributable to dietary intake 10–15 years ago would be greater than those based on the current diet. Taken together, the cancer burden associated with suboptimal diet among US adults may be greater than the current estimates.

In 2015, more than 80 000 new cancer cases among US adults were associated with suboptimal dietary intake. Highest cancer burden was associated with insufficient whole grains and excess processed meats. Middle-aged men and racial/ethnic minorities experienced the largest proportion of diet-associated cancer burden. Our findings underscore the need for reducing cancer burden and disparity in the United States by improving the intake of key food groups and nutrients of Americans.

## Funding

This work was supported by NIH/NIMHD 1R01MD011501 (FFZ), NIH/NHLBI R01HL115189 (DM), United Kingdom Medical Research Council Epidemiology Unit Core Support (MC_UU_12015/5) (FI), and American Heart Association postdoctoral fellowship (JXL).

## Notes

Affiliations of authors: Friedman School of Nutrition Science and Policy (FFZ, FC, ZS, HE, JL, MD, LL, PW, DM) and School of Medicine (DSMMR), Tufts University, Boston, MA; T. H. Chan School of Public Health, Harvard University, Boston, MA (ZS); MRC Epidemiology Unit, University of Cambridge, Cambridge, UK (FI); Department of Epidemiology and Population Health, Albert Einstein College of Medicine, Bronx, NY (CDR); Institute for Clinical Research and Health Policy Studies, Tufts Medical Center, Boston, MA (DK).

Dr Mozaffarian reports honoraria or consulting from AstraZeneca, Acasti Pharma, GOED, Haas Avocado Board, Nutrition Impact, Pollock Communications, Boston Heart Diagnostics, and Bunge; scientific advisory board, Omada Health and Elysium Health; chapter royalties from UpToDate; and research funding from National Institutes for Health and Gates Foundation.

Dr Zhang had full access to all the data in the study and takes responsibility for the integrity of the data and the accuracy of the data analysis. Drs Cudhea, Shan, and Rehm and Ms Eom and Ms Ruan conducted the data analysis.

The funding sources had no role in the design and conduct of the study; collection, management, analysis, and interpretation of the data; preparation, review, or approval of the manuscript; and decision to submit the manuscript for publication.

## Supplementary Material

Supplementary_Material_pkz034
