# Supplementary material for: Preventable Cancer Burden Associated With Poor Diet in the United States
Source: JNCI Cancer Spectr. 2019 May 22;3(2):pkz034. doi: 10.1093/jncics/pkz034 (PMC6649723; doi:10.1093/jncics/pkz034)
Supplement: Supplementary_Material_pkz034 [file supplementary_material_pkz034.pdf]

Supplemental Online Content

Table of Contents

Appendix 3
Appendix 1. Comparative Risk Assessment 3
Population Attributable Fraction 3
Computation 6
Joint PAF 7
Monte Carlo Simulations 8
Population Attributable Fraction via Mediated Effects 8
Appendix 2. Etiologic Relationships of Dietary Factors with Cancer Risk 11
Study Designs Used for RR Estimates 12
Heterogeneity of RR Estimates by Population Subgroups 13
Appendix 3. De Novo Meta-Analyses of Fruit and Vegetable Intake and Incidence and Mortality of Mouth, Pharynx and Larynx Cancer 14
Text A. Protocol for De Novo Meta-Analysis of Fruit and Vegetable Intake and Risk of Mouth, Pharynx, and Larynx cancer 14
Text B. Search Results of Prospective Cohort Studies Examining the Fruit/Vegetable Intake and Risk of Mouth, Pharynx, and Larynx Cancer 21
Text C. Meta-Analysis Results of Fruit and Vegetable Intake and Risk of Mouth, Pharynx, and Larynx Cancer 22
Appendix 4. Number of New Cancer Cases in the US by Age, Sex, and Race/Ethnicity . **25**

Supplementary Tables 26
Table 1. Relative Risk Estimates of Etiologic Relationships between Diet and Cancer 26
Table 2. Relative Risk Estimates of Etiologic Relationships between Body Mass Index and Cancer 27
Table 3. Pooled Multivariable-Adjusted Relationships of Changes in Dietary Habits with Change in BMI among 120,977 U.S. Woman and Men in Three Prospective Cohorts with 12-20 Years Follow-up[1] 28
Table 4. Estimated Number and Proportion (%) of New Cancer Cases Associated with Suboptimal Dietary Intake Among US Adults in 2015, by Cancer Site and Dietary Target 29
Table 5. Estimated Number and Proportion of New Cancer Cases Associated with Suboptimal Dietary Intake Among US Adults in 2015, by Cancer Site and Population Subgroups 32

Table 6. Estimated Number of New Cancer Cases Associated with Suboptimal Dietary Intake Among US Adults, by Dietary Factor and Population Subgroups 37

Supplementary Figures 40

Figure 1. Proportion of New Cancer Cases (%) Associated with Suboptimal Diet among U.S. Adults in 2015, by Cancer Type 40

Figure 2. Proportion of New Cancer Cases (%) Associated with Suboptimal Diet Among U.S. Adults in 2015, by Dietary Factors 41

Supplementary References 42

Appendix

Appendix 1. Comparative Risk Assessment

Population Attributable Fraction

A Comparative Risk Assessment (CRA) framework has been used to estimate the proportion of cancer cases attributable to suboptimal diet, the Population Attributable Fraction (PAF). The standard PAF formula used is as follows:

$$\frac{\int_{x=0}^{m} RR(x)P(x)dx - 1}{\int_{x=0}^{m} RR(x)P(x)dx}$$

where $P(x)$ is the distribution of current dietary consumption, $RR(x)$ is the relative risk of mortality at exposure level x, and m is the maximum exposure level. As described by Vander Hoorn et al,[1,2] this is a special case of the more commonly used (for descriptive purposes) formula:

$$\frac{\int_{x=0}^{m} RR(x)P(x)dx - \int_{x=0}^{m} RR(x)P'(x)dx}{\int_{x=0}^{m} RR(x)P(x)dx}$$

where the alternative distribution ($P'(x)$) is the same as the theoretical minimum risk (optimal population distribution of diet) exposure distribution.

$\boldsymbol{P(x)}$

We estimated the mean intake and distribution of dietary intake of seven food groups and nutrients among US adults aged 20 years or older using the dietary data collected from 24-diet recalls in the National Health and Nutrition Examination Survey (NHANES). Dietary data from one or two 24-hour diet recalls may not represent a person's usual intake due to within-person

variations in food intake. To correct for measurement errors, we applied the National Cancer Institute (NCI) method to estimate usual intake of nutrients from foods.[3] As documented in prior literature, the NCI method is the preferred method for estimating usual intake distribution from 24-hour diet recalls.[4] A 2-step approach was used to estimate usual intake in the NCI method. The first step (MIXTRAN macro) models the amount of a daily-consumed dietary factor but both the amount and probability of an episodically-consumed dietary factor. For the dietary factors that are episodically consumed with more than 5% of the individuals reporting zero intake on a given day such as fruits, non-starchy vegetables, whole grains, processed meats, unprocessed red meats, sugar-sweetened beverages (SSBs), we used a two-part model that estimates both the amount and probability of consumptionday.[4] The second step of the NCI method involves estimating usual intake with parameters estimated from the first step using mixed-effect linear regression on a transformed scale with a person-specific effect (INDIVINT macro).[3] The NCI method requires that some of the participants have multiple days of nutrient intake to estimate and separate the within and between-person variations.[5] In our study, 8683 also provided a second valid diet recall (86% of the 10064 participants who provided first valid recall). For each nutrient, the following covariates were specified in estimating usual intake: age group (20-34, 35-49, 50-64, 65+ years), sex, race/ethnicity (non-Hispanic white, non-Hispanic black, Hispanic, and other), an indicator of first *versus* second-day diet recall, and day of the week when recall occurs (weekday *versus* weekend). We further incorporated the weights from the complex survey sample design to estimate the mean intake and distribution of the dietary factors and nutrients included in this study.

Because distributions of many dietary factors are non-normal, we utilized a gamma distribution for each factor after confirming, using individual-level NHANES data, that this estimation is similar to the normal distribution for normally distributed dietary factors and closer to observed data for skewed dietary factors than normal or log-normal distributions; and that the gamma distribution also performs optimally for estimating population attributable fractions. Specifically, based on a visual inspection of histograms, we concluded that, overall, the gamma distribution fit the NHANES data better than an alternative right-skewed distribution (the log-normal), particularly for foods where the intake is highly skewed, such as processed meat and sugar-sweetened beverages. Simulations done to compare estimated attributable mortality estimates assuming gamma, normal, and log-normal distributions to mortality estimates based on a non-parametric approach showed that estimates assuming the gamma distribution gave closer estimates to the non-parametric approach than the others. Because the mean and variance of the gamma distribution is a function of the parameters of the gamma distribution ($E[X] = \frac{\alpha}{\beta}$, $Var[X] = \frac{\alpha}{\beta^2}$ where X is a gamma random variable, α is the shape parameter and β is the scale parameter), estimates for the gamma parameter can be obtained from mean and variance estimates that account for survey design characteristics.

$\boldsymbol{RR(x)}$

$RR(x)$ is defined to be

$$\begin{cases} exp(\beta(x - y(x))) & : x - y(x) \geq 0 \\ 1 & : x - y(x) < 0 \end{cases}$$

where β is the the change in log relative risk per unit of exposure, x is the current exposure level, and $y(x)$ is the optimal (theoretical minimum risk, TMRED) exposure level. $y(x)$ is defined to be $F_{TMRED}(F_X^{-1}(x))$, where F_{TMRED} is the cumulative distribution function of the TMRED and F_X^{-1} is the inverse cumulative distribution function of the current exposure distribution. Implicit in how we characterize the relative risk function are some of the fundamental assumptions we make about relative risk. Namely, that relative risk increases exponentially as distance from theoretical minimum risk exposure level (y) increases, that there is no risk associated with exposure beyond the theoretical minimum risk exposure level, and that both x and the theoretical minimum risk exposure level for an individual at exposure level x are the q-th quantile of their respective distributions (the observed exposure distribution, and the TMRED, respectively). TMRED was characterized based on the optimal distribution associated with lowest disease risk, assessed by the Global Burden of Disease (GBD) 2010 with three considerations: the availability of strong evidence that supports a continuous reduction in cancer risk to optimal intake; the distribution that is feasible at the population level; and consistency with major dietary guidelines.[6]

$\boldsymbol{m}$

In our analyses, m is defined to be ∞. Since the density of a gamma distribution approaches 0 as exposure, x, approaches infinity, and because implausibly high values of exposure should exceed the corresponding theoretical maximum exposure level, implausibly high values of exposure will make little to no contributions to the PAF.

Computation

In practice, we use simple numerical integration (using Riemann sums) to compute the integrals in the PAF formula. Thus, we used the categorical equivalent of the PAF formula

$$PAF = \frac{\sum_{i=1}^{n} P_i \,(RR_i - 1)}{\sum_{i=1}^{n} P_i \,(RR_i - 1) + 1}$$

where the n categories are determined by dividing up the exposure range (chosen here to be $[0, F_X^{-1}(\Phi(6))]$) into 121 intervals, each of length 0.1 when converted to the standard normal scale (except for the first one). More precisely, the range of exposure group i can be described as follows:

$$\begin{array}{ll} [0, F_X^{-1}(\Phi(-6))] & : i = 1 \\ (F_X^{-1}(\Phi(-6 + 0.1(i-2))), F_X^{-1}(\Phi(-6 + 0.1(i-1)))] & : i > 1 \end{array}$$

Joint PAF

Because summing would overestimate joint relationships,[6] for each stratum and cancer site, the joint PAF of overall suboptimal diet was estimated by proportional multiplication:

$$PAF_{joint} = 1 - \prod_{r=1}^{R}(1 - PAF_r)$$

where r denotes each individual dietary factor, and R is the number of dietary factors. The analyses supported independent etiologic relationships of each dietary factor, and joint distributions were further determined within each stratum; maximizing validity of our joint PAFs. Joint distributions of exposure may be partly correlated among individuals, leading to overestimation of joint attributable fractions. Separate validity analyses of dietary pattern studies done by others suggested that the estimated etiologic relationships of individual components and their joint associations were each reasonable.[7]

Monte Carlo Simulations

Monte Carlo simulations were used to quantify uncertainty in the PAFs, incorporating uncertainty in dietary exposure distributions, etiologic RR estimates, and for BMI-mediated associations, prevalence of overweight/obesity. Specifically, for each diet disease pair and stratum, we drew randomly 1,000 times from the normal distribution of the estimate of disease-specific change in the log(RR) corresponding to a one-unit increase in intake, the normal distribution of the estimate of the exposure mean, and where appropriate, the normal distribution of the estimate of the prevalence of obesity. Draws of proportions that were less than 0 or greater than 1 were changed to 0 or 1, respectively. Likewise, draws of mean intake that were zero or less were changed to 0.00001. Each set of random draws was used to calculate the PAFs and associated cancer incidence.

Population Attributable Fraction via Mediated Effects

In order to estimate diet-related cancer burden mediated by obesity, we associated the effect of changes in dietary factors on change in BMI (the diet-BMI effect size) with the association of BMI with cancer risk (the BMI-cancer RR). Specifically, we estimated log(RR) of cancer risk associated per unit (kg/m^2) increase in BMI by taking the log(RR) of cancer risk associated per unit increase in BMI and multiplying it by an estimated effect size of BMI associated with per unit increase in dietary exposure; and the latter for a given subgroup was a weighted average of the effect on BMI for overweight (BMI $\geq$ 25) individuals and non-overweight (BMI $<$ 25) individuals, with the weights determined by the prevalence of overweight for that subgroup. Similar approaches have been applied to estimate the effect of sodium intake on cardiovascular

diseases (CVDs) by combining the effect of high sodium intake on blood pressure and the effect of high pressure on CVDs.[8]

The diet-BMI effect size was estimated based on multivariable-adjusted results pooled from 120,877 US men and women in three separate prospective cohort studies: 50,422 women in the Nurses' Health Study, 47,898 women in the Nurses' Health Study 2, and 22,557 men in the Health Professionals Follow-up Study that were followed at 4-year intervals over 12-20 years.[9] Separate linear relationships were estimated for individuals with BMI <25 and BMI ≥ 25 since the rate of associated increase in BMI due to these dietary factors varies based on an individual's baseline BMI.

The BMI-cancer RR estimates were obtained from the meta-analysis conducted by IARC in 2016[10] and in the series of CUP reports by WCRF/AICR (eTable 3 in the Supplement). Thirteen cancers (endometrial, esophageal adenocarcinoma, kidney, liver, gallbladder, stomach cardia, postmenopausal breast, pancreatic, advanced prostate, ovarian and colorectal cancers) were associated with body fatness evaluated by WCRF/AICR with evidence grading as “convincing” or “probable”. We included two additional cancer types (thyroid cancer and multiple myeloma) associated with body fatness evaluated by IARC for with evidence grading as “sufficient”, for a total of 15 cancers associated with body fatness (BMI).

For SSB, current evidence does not support a direct association between SSB consumption and cancer risk.[11,12] Thus, the total cancer burden attributed to high SSB intake reflect entirely the

BMI-mediated associations. For fruits, vegetables, whole grains, red meats, and processed meats, direct associations with cancer risk (after adjustment for BMI) were included; and the total cancer burden attributable to these dietary factors was a combination of cancer burden attributable to direct associations and that attributable to BMI-mediated associations.

Appendix 2. Etiologic Relationships of Dietary Factors with Cancer Risk

Our selection of dietary factors and corresponding relative risk (RR)'s was informed by the World Cancer Research Fund (WCRF)/American Institute for Cancer Research (AICR) reports on the RR estimates of dietary factors with cancer incidence and mortality. The WCRF/AICR have performed Systematic Literature Review (SLR) to evaluate the evidence of various dietary factors on cancer incidence and mortality, with the Second Expert Report published in 2007 and the Continuous Update Project (CUP) Expert Report published in 2018.[13] The strength of evidence is categorized into "convincing", "probable", "limited-suggestive", "limited-no conclusion", and "substantial effect unlikely" (eTable 2 in the Supplement). Based on the WCRF/AICR grading system, we focused on 7 dietary targets having "convincing" or "probable" evidence for effects on cancer risk: fruits, non-starchy vegetables, whole grains, processed meats, red meats, whole grains, total dairy products, and sugar-sweetened beverages (SSBs) (Table 1). The present analysis incorporated the RR estimates from meta-analyses of prospective cohort studies with limited evidence of bias from confounding, such as the RR estimates evaluated by WCRF/AICR on whole grains, processed meats, and red meats in association with risks of colorectal cancer[14] and stomach cancer (non-cardia).[15] Both whole grains and foods high in fiber decrease the risk of colorectal cancer,[14] and whole grain foods are an important source of dietary fiber. Similarly, both dairy products and foods high in calcium decrease the risk of colorectal cancer, [14] and dairy products are an important source of calcium. We included whole grains and total dairy products but not fiber and calcium in the same analysis to avoid overestimation. SSB was not quantified as a separate food group in WCRF/AICR CUP reports for cancer risk. However, its causal impact on adiposity[16] provides strong support to include SSB as a dietary target for estimating cancer burden (eAppendix 1).[17-19]

When RR estimates were not directly available from the WCRF/AICR reports for some cancers (mouth, pharynx, and larynx cancers) or dietary target (SSB), we performed systematic searches on PubMed to identify meta-analyses that evaluated these specific cancer types and dietary targets; or when eligible meta-analysis was not available, we conducted *de novo* meta-analyses of prospective cohort studies to estimate the RR (eAppendix 3 in the Supplement). Published meta-analyses were eligible if including randomized trials or prospective cohort studies of the identified diet-disease relationships of interest. Whenever possible, we prioritized meta-analyses that characterized dose-responses using all available data (as opposed to comparisons of extreme categories, e.g., highest vs. lowest quartiles). Meta-analysis including only retrospective case-control studies were excluded due to greater potential for selection bias, recall bias, and reverse causation. When more than one meta-analysis was identified for any diet-disease relationship, we included the dose-response estimates with the greatest number of studies and clinical events. When meta-analysis based on randomized trials or prospective cohort studies was not available from WCRF/AICR reports, CUP, or PubMed systematic searchers, we developed our own systematic review protocols and performed de novo meta-analyses (e.g., for mouth, pharynx, and larynx cancers).

Study Designs Used for RR Estimates

Both prospective cohort studies and randomized controlled trials (RCTs) provide important evidence for identification of effects of dietary factors on cancer risk. However, RRs for diet and cancer risk came mostly from prospective cohort studies not RCTs.[20] Very few large-scale RCTs have been conducted to evaluate dietary intake of foods and nutrients directly on cancer incidence (i.e. Women's Health Initiative Dietary Modification trial for reducing dietary fat

intake and increasing consumption of fruits and vegetables). Some RCTs (e.g., SELECT, Physician's Health Study II RCT, ATBC, VITAL) have focused on vitamins or minerals in the supplemental form. The lack of evidence from RCTs directly assessing diet and cancer risk reflects the complex nature of dietary exposure and cancer etiology. Different from a linear drug trial, the long latency period for dietary exposures to cause cancer requires participants adhering to dietary interventions for extended follow-up, often in decades, making RCTs impractical and prohibitive. High dropout rates, highly selective study population, and different forms of dietary factors complicate the interpretation and diminish the feasible use of RCTs.[20,21] Therefore, WCRF/AICR reports explicitly note that although RCTs are important, results of these should not automatically override evidence from other type of studies. This resulted in placing the greatest emphasis on prospective cohort studies or RCTs "where available and appropriate" when assigning evidence categories.[22] Thus, the RR estimates for diet and cancer risk are based on long-term prospective cohort studies that have adjusted for major confounders and, when available, for bias introduced by measurement error in the exposure[23] that generally produces underestimation of the true RR.[24] Notably, this approach has also been used for estimating the effects of smoking, alcohol, adiposity, and physical inactivity on cancer risk.

Heterogeneity of RR Estimates by Population Subgroups

In WCRF reports and our work to-date, the current evidence for diet and cancer risk, both from individual studies and meta-analyses, suggests that when measured comparably, the proportional effects considered in this proposal are similar across different age, sex, and racial/ethnic groups. Because substantial interactions of age, sex, or race/ethnicity in these RR estimates are not observed, homogeneous RR estimates were applied in population subgroups.

Appendix 3. De Novo Meta-Analyses of Fruit and Vegetable Intake and Incidence and Mortality of Mouth, Pharynx and Larynx Cancer

Text A. Protocol for De Novo Meta-Analysis of Fruit and Vegetable Intake and Risk of Mouth, Pharynx, and Larynx cancer

Objective

To systematically review and summarize the evidence from prospective cohort studies and/or randomized controlled trials on the association between fruit and vegetable intake and incidence and mortality of cancer in the mouth, pharynx and larynx in men and women.

Methods

We adapted the systematic review protocol of the WCRF/AICR CUP reports for cancers of mouth, pharynx and larynx,[25] following the recommendations of Preferred Reporting Items for Systematic reviews and Meta-Analyses (PRISMA) and Meta-analysis of Observational Studies in Epidemiology (MOOSE) guidelines during all stages of the design, implementation, and reporting of this meta-analysis.

Inclusion criteria

- **Study designs:** Randomized controlled trials or prospective cohort studies (including nested case-control design)
- **Study population:** Men, women or both 18 years or older

- **Exposure:** Intake of fruits and vegetables, either continuous or in >2 categories of intake to allow for adequate categorization of fruit and vegetable intake. Exposure must refer to a period before cancer diagnosis.
- **Outcome:** Cancers of the mouth, pharynx, and larynx as separate outcomes, as combinations of these cancers, or for all these cancers combined[a]
- **Effect estimate:** Studies providing multi-variate adjusted effect estimate and variance and information on, or sufficient information to calculate effect estimate variance.
- **Year:** Published in MEDLINE from January 1st, 2006[b]
- **Language:** English
- **Publication type:** Full-text, published, peer reviewed.

a. Cancers of the mouth and pharynx include those of the tongue, gums and floor and other parts of the mouth, and the pharynx. Articles identified in the search with the following outcomes: "head and neck cancer", "upper aero-digestive cancers" and other cancers groups that explicitly include mouth, pharynx or larynx cancers will also be extracted.

b. January 1st, 2006 is the closure date of the database for the WCRF/AICR Second Expert Report. We will perform de novo searches only for studies published after WCRF/AICR Second Export Report but will include prospective studies that have been identified at the WCRF/AICR Second Expert Report that were published prior to January 1st, 2006 into meta-analysis

Exclusion criteria

- Case-control studies, cross-sectional studies, etiologic studies

- Studies in which the only measure of the relationship between the relevant exposure and outcome is the mean difference of exposure (this is because the difference is not adjusted for main confounders).
- Studies in which the major outcome include esophageal cancer.
- Studies in which the outcome is exclusively cancer of the lip and/or salivary glands.
- Study results in which the exposure is a biomarker taken at or after cancer diagnosis.
- Articles written in language other than English, French, Spanish, Portuguese or Italian, if it is not possible to obtain a translation of the article.

Search database

The MEDLINE database will be searched using PubMed as platform. Hand search will be performed for the references of reviews and meta-analyses identified during the search.

Search strategy

Limits:

Age: Any

Setting: Any country

Year Range: From 2006/01/01 to 2017/06/30

Language: English

Species: Human

Search Terms:

EXPOSURE

diet therapy[MeSH Terms] OR nutrition [MeSH Terms] OR diet [Title/Abstract] OR diets [Title/Abstract] OR dietetic [Title/Abstract] OR dietary [Title/Abstract] OR eating [Title/Abstract] OR intake [Title/Abstract] OR nutrient*[Title/Abstract] OR nutrition [Title/Abstract] OR vegeterian*[Title/Abstract] OR vegan*[Title/Abstract] OR "seventh day adventist" [Title/Abstract] OR macrobiotic [Title/Abstract] OR food and beverages [MeSH Terms] OR food* [Title/Abstract] OR vegetable* [Title/Abstract] OR fruit* [Title/Abstract] OR legume* [Title/Abstract] OR soy [Title/Abstract] OR soya [Title/Abstract] OR nu t[Title/Abstract] OR nuts [Title/Abstract] OR peanut*[Title/Abstract] OR food preservation [MeSH Terms]

OUTCOME

Larynx [Title/Abstract] OR pharynx [Title/Abstract] OR laryngeal [Title/Abstract] OR pharyngeal [Title/Abstract] OR hypopharyngeal [Title/Abstract] OR oropharyngeal [Title/Abstract] OR mouth [Title/Abstract] OR tongue[Title/Abstract]

AND

malign*[Title/Abstract] OR cancer*[Title/Abstract] OR carcinoma*[Title/Abstract] OR tumor*[Title/Abstract] OR tumour*[Title/Abstract] OR adenocarcinoma*[Title/Abstract] OR carcinoma, squamous cell*[Title/Abstract] OR carcinoma, small cell*[Title/Abstract])

OR

Laryngeal Neoplasms [MeSH Terms] OR Pharyngeal Neoplasms [MeSH Terms] OR Mouth Neoplasms [MeSH Terms] OR Tongue Neoplasms [MeSH Terms] OR Oropharyngeal Neoplasms [MeSH Terms] OR Hypopharyngeal Neoplasms [MeSH Terms]) OR Oral

Leukoplakia [MeSH Terms] OR oral cancer*[Title/Abstract] OR oral carcinoma*[Title/Abstract] OR oral leukoplakia*[Title/Abstract]

Article selection

First, all references obtained with the searches in PubMed will be imported in the reference manager database "Abstrackr".[26] Second, two reviewers will independently screen the titles and abstracts of all references. Third, the reviewers will assess the full manuscripts of all papers for which eligibility could not be determined by reading the title and abstract. The reviewers will solve any disagreements about the study or exposure relevance by discussion with the principal investigator.

Data extraction

The data to be extracted include among others, the study design, name, characteristics of study population, age range, sex, country, recruitment year, methods of exposure assessment, definition of exposure, definition of outcome, method of outcome assessment, study size, number of cases, number of comparison subjects, length of follow up, lost to follow-up, analytical methods and whether methods for correction of measurement error were used.

The ranges, means or median values for each exposure level will be extracted as reported in the paper. The reviewer will not do any calculation during data extraction. For each result, the reviewers will extract the covariates and matching variables included in the analytical models. Measures of association, number of cases and person years for each category of exposure will be extracted for each analytical model reported.

Some studies present results for the cancers of interest as separate outcomes (mouth, pharynx and larynx), combinations of these cancers, or total results for these cancers. In some cases, esophageal and nasopharyngeal cases may also be included. The reviewer will extract the results for each cancer site and for the cancer groups relevant to the review. The reviewer will also extract the results by sex, age group, race/ethnicity and other subgroups, if reported, and for combined results when presented in the paper. The data extracted will be double-checked by a second reviewer.

Meta-analysis

Dose-response meta-analysis will be conducted to express the results of each study in the same increment unit for a given exposure, using the "best" adjusted models. The best adjusted model will usually be the most adjusted model. When the linear dose-response estimate is reported in an article, this will be used in the dose-response meta-analysis. If the results are presented only for categorical exposures/intervention (quantiles or pre-defined categories), the slope of the dose-response relationship for each study will be derived from the categorical data using generalized least-squares for trend estimation (command GLST in Stata).[27] The meta-analysis results will be shown in a dose-response forest plots. For comparability, the same increment units used in the meta-analyses of WCRF/AICR CUPs (e.g., servings/d) will be used to present the linear dose-response analysis results.

If the dose response estimates are not reported in an article, this will be derived from categorical data This method accounts for the correlation between relative risks estimates with respect to the

same reference category.[28] The dose-response model is forcing the fitted line to go through the origin and whenever the assigned dose corresponding to the reference group (RR=1) is different from zero, this will be rescaled to zero and the assigned doses to the other exposure categories will be rescaled accordingly.

Heterogeneity between studies will be quantified with the I^2 statistic with cut points for I^2 values of 30%, and 50% for low, moderate, and high degrees of heterogeneity.[29] Heterogeneity will be assessed visually from forest plots and with statistical tests (P value <0.05 will be considered statistically significant) but the interpretation will rely mainly in the I^2 values as the test has low power and the number of studies will probably be low.

Text B. Search Results of Prospective Cohort Studies Examining the Fruit/Vegetable Intake and Risk of Mouth, Pharynx, and Larynx Cancer

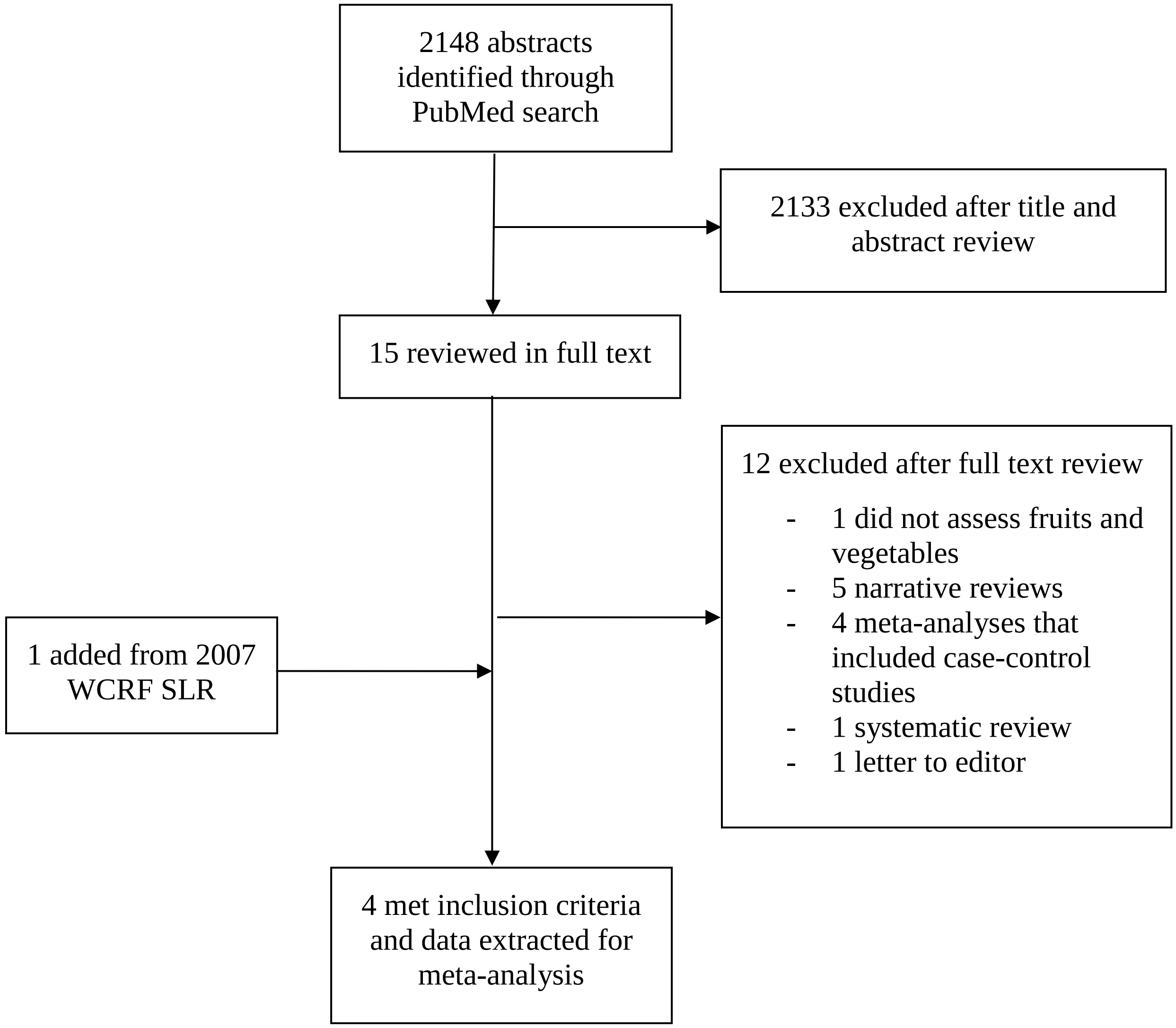

Text C. Meta-Analysis Results of Fruit and Vegetable Intake and Risk of Mouth, Pharynx, and Larynx Cancer

C-1. Forest Plot for Dose-Response Meta-Analysis: Fruit and Vegetable Intake and Risk of Mouth, Pharynx, and Larynx Cancer, Prospective Cohort Studies[30-32]

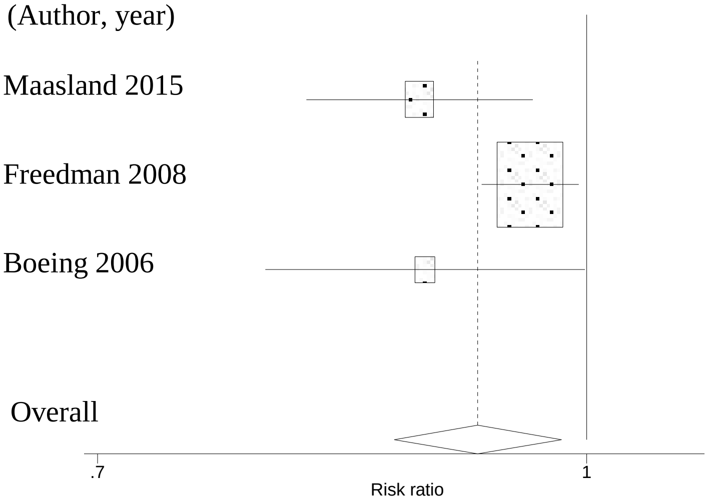

**Study** (Author, year)	**Vegetables and Fruits** Relative Risk (95% CI)	**% Weight**
Maasland 2015	0.89 (0.82 to 0.96)	29.20
Freedman 2008	0.96 (0.93 to 0.99)	51.74
Boeing 2006	0.89 (0.79 to 1.00)	19.06
Overall	0.92 (0.87 to 0.98)	100

Test for heterogeneity: I^2*=52.06% (p=0.12)*

C-2. Forest Plot for Dose-Response Meta-Analysis: Fruit Intake and Risk of Mouth, Pharynx, and Larynx Cancer, Prospective Cohort Studies[30-33]

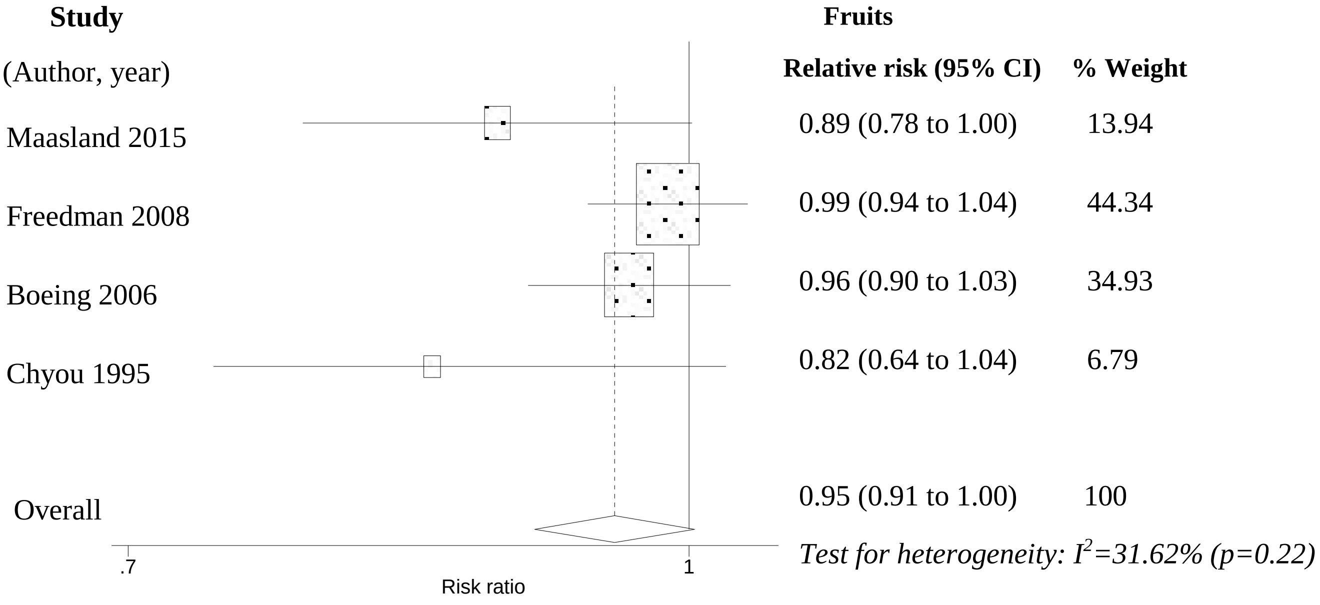

C-3. Forest Plot for Dose-Response Meta-Analysis: Vegetable Intake and Risk of Mouth, Pharynx, and Larynx Cancer, Prospective Cohort Studies[30-32]

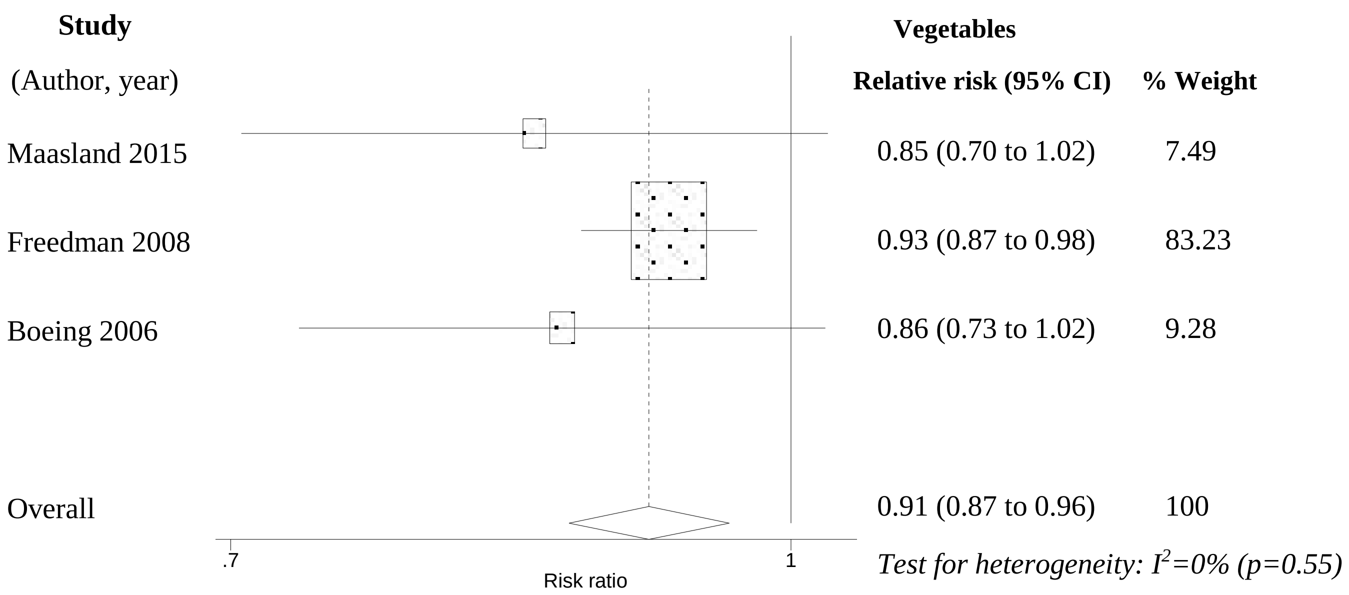

Appendix 4. Number of New Cancer Cases in the US by Age, Sex, and Race/Ethnicity

The most recently reported cancer statistics (2015) in US adults and by population subgroups were obtained from the Centers for Disease Control and Prevention's (CDC's) National Program for Cancer Registries (NPCR) and the National Cancer Institute's (NCI's) Surveillance, Epidemiology, and End Results (SEER) program, which collectively provided complete number of cancer cases for the US population. Cases for individual cancer types were obtained by applying the International Classification for Diseases for Oncology, third edition (ICD-O-3) codes corresponding to primary cancer site. Additional specifications on tumor histologic types and anatomic locations were used to obtain the incident cancer cases for esophageal adenocarcinoma and stomach cardia and non-cardia cancers. In the 2015 WCRF/AICR CUP reports, advanced prostate cancer was defined as cancers reported in any of the following ways: stage 3-4 on the American Joint Committee on Cancer (AJCC) 1992 classification; advanced or metastatic cancer; metastatic cancer; stage C or D on the Whitmore/Jewett scale; fatal cancer (prostate specific mortality); high stage or grade; or Gleason grade ≥ 7. Per this definition, approximately 17% of all prostate cancer cases were regional and distant cancers at diagnosis.[34] We applied this proportion to all new prostate cancer cases to estimate the number of advanced prostate cancer cases. We included new breast cancer cases diagnosed at ages 51 years or older to estimate the number of post-menopausal breast cancer cases.

Supplementary Tables

Table 1. Relative Risk Estimates of Etiologic Relationships between Diet and Cancer

Dietary Factors	Cancer	No. of Studies (No. of Events) in each meta-analysis	Source	Evidence Grading[1]	Unit of RR	RR (95% CI) per unit of RR	Statistical Heterogeneity
Fruits	Mouth, pharynx, and larynx	4 (1,646)	*de novo* meta-analysis	Probable ↓ risk	100g /d	0.95 (0.91, 1.00)	I^2=31.6%, P=0.22
Vegetables	Mouth, pharynx, and larynx	3 (1,554)	*de novo* meta-analysis	Probable ↓ risk	100g /d	0.91 (0.87, 0.96)	I^2=0, P=0.55
Whole Grains	Colon and rectum	6 (8,320)	CUP, 2018	Probable ↓ risk	90g/d	0.83 (0.78, 0.89)	I^2=18%, P=0.30
Processed Meats	Colon and rectum	10 (10,738)	CUP, 2018	Convincing ↑risk	50g/d	1.16 (1.08, 1.26)	I^2=20%, P=0.26
	Stomach (non-cardia)	3 (1,149)	CUP, 2018	Probable ↑ risk	50g/d	1.18 (1.01.1.38)	I^2=3.2%, P=0.36
Red Meats	Colon and rectum	8 (6,662)	CUP, 2018	Probable ↑risk	100g/d	1.12 (1.00, 1.25)	I^2=24%, P=0.24
Total Dairy products	Colon and rectum	10 (13,224)	CUP, 2018	Probable ↓risk	400g/d	0.87 (0.83, 0.90)	I^2=18%, P=0.27

Abbreviations: RR, Relative Risk

1.Evidence grading and RR estimates were based on the WCRF/AICR 2018 Continuous Update Project (CUP) Expert Report for most cancers (eAppendix 2).[13] Evidence grading and RR estimates for cancers in mouth, pharynx and larynx were based on *de novo* meta-analysis of prospective cohort studies (eAppendix 3).

Table 2. Relative Risk Estimates of Etiologic Relationships between Body Mass Index and Cancer

Cancer Type	No. of Studies	No. of Events	Source	Evidence Grading[1]	RR (95% CI) Per 5 kg/m^2	Statistical Heterogeneity
Corpus uteri	26	18,717	CUP, 2018	Convincing ↑risk	1.50 (1.42-1.59)	I^2=86.2% P<0.0001
Esophageal (adenocarcinoma)	9	1,725	CUP, 2018	Convincing ↑risk	1.48 (1.35-1.62)	I^2=36.7% P=0.13
Kidney	23	15,575	CUP, 2018	Convincing ↑risk	1.30 (1.25-1.35)	I^2=38.8% P=0.03
Liver	12	14, 311	CUP, 2018	Convincing ↑risk	1.30 (1.16-1.46)	I^2=78.3% P=0.000
Gallbladder	8	6,004	CUP, 2018	Probable ↑risk	1.25 (1.15-1.37)	I^2=52.3% P=0.04
Stomach (cardia)	7	2,050	CUP, 2018	Probable ↑risk	1.23 (1.07-1.40)	I^2=55.6% P=0.04
Breast (post-menopausal)	56	80,404	CUP, 2018	Convincing ↑risk	1.12 (1.09-1.15)	I^2=75% P<0.001
Pancreas	23	9,504	CUP, 2018	Convincing ↑risk	1.10 (1.07-1.14)	I^2=19% P=0.20
Multiple myeloma	20	1,388	IARC, 2016	Sufficient (IRAC) ↑risk	1.09 (1.03-1.16)	Not reported
Prostate (advanced)	24	11,149	CUP, 2018	Probable ↑risk	1.08 (1.04-1.12)	I^2=18.8% P=0.21
Thyroid	22	3,100	IARC, 2016	Sufficient (IARC) ↑risk	1.06 (1.02-1.10)	Not reported
Ovary	25	15,899	CUP, 2018	Probable ↑risk	1.06 (1.02-1.11)	I^2=55.1% P=0.001
Colorectal	38	71,089	CUP, 2018	Convincing ↑risk	1.05 (1.03-1.07)	I^2=74.2% P=0.000

Abbreviations: RR, Relative Risk

1. Evidence grading and RR estimates were based on the WCRF/AICR 2018 Continuous Update Project (CUP) Expert Report for most cancers (eAppendix 2).[13] For multiple myeloma and thyroid cancer, evidence grading and RR estimates were based on the meta-analysis conducted by IARC in 2016.[10]

Table 3. Pooled Multivariable-Adjusted Relationships of Changes in Dietary Habits with Change in BMI among 120,977 U.S. Woman and Men in Three Prospective Cohorts with 12-20 Years Follow-up[1]

Dietary Targets, serving/d	Multivariable-Adjusted Change in BMI (95% CI),[2] kg/m^2					
	All participants	P value	Participants with BMI < 25 kg/m^2	P value	Participants with BMI ≥25 kg/m^2	P value
Fruits	-0.08 (-0.11, -0.05)	<0.001	-0.06 (-0.08, -0.04)	<0.001	-0.11 (-0.16, -0.06)	<0.001
Non-starch vegetables	-0.04 (-0.06, -0.02)	<0.001	-0.03 (-0.04, -0.01)	<0.001	-0.06 (-0.09, -0.02)	0.001
Whole grains [3]	-0.06 (-0.08, -0.04)	<0.001	-0.05 (-0.07, -0.03)	<0.001	-0.08 (-0.10, -0.06)	<0.001
Processed meats	0.16 (0.11, 0.10)	<0.001	0.13 (0.07-0.19)	<0.001	0.16 (0.11, 0.21)	<0.001
Unprocessed red meats	0.17 (0.11, 0.23)	<0.001	0.13 (0.07, 0.20)	<0.001	0.23 (0.14, 0.32)	<0.001
Sugar sweetened beverages	0.14 (0.09, 0.19)	<0.001	0.09 (0.05, 0.14)	<0.001	0.23 (0.14, 0.32)	<0.001

Abbreviation: BMI, body mass index

1. Based on pooled results from 3 separate prospective cohort studies, including 50,422 women in the Nurses' Health Study (1986-2006), 47,898 women in the Nurses Health Study2 (1991-2003), and 22,557 men in the Health Professionals Follow-up Study (1986-2006) who were free of obesity or chronic diseases and with complete data on weight and lifestyle habits at baseline. Women who became pregnant during follow-up were excluded from the analysis. Independent relations of changes in dietary habits with BMI change were assessed in 4-year periods over 20 years in the Nurses' Health Study, 12 years in the Nurses Health Study2, and 20 years in the Health Professionals Follow-up Study, using linear regression with robust variance and accounting for within-person repeated measures.
2. BMI changes shown are for increased consumption; decreased consumption would be associated with the inverse BMI change. All results are adjusted for all of the dietary factors in the Table simultaneously as well as for age, baseline body mass index at the beginning of each 4-year period, sleep duration, and changes in physical activity, alcohol use, television watching, and smoking.
3. Findings were similar when either total dietary fiber or cereal fiber were evaluated in the analysis instead of whole grains.

Table 4. Estimated Number and Proportion (%) of New Cancer Cases Associated with Suboptimal Dietary Intake Among US Adults in 2015, by Cancer Site and Dietary Target

Annual Cancer Burden Attributable to Suboptimal Dietary Intake	Total Cancer Burden		Direct Cancer Burden		Obesity-Mediated Cancer Burden	
	Incidence N (95% UI)	PAF % (95 UI)	Incidence N (95% UI)	PAF % (95 UI)	Incidence N (95% UI)	PAF % (95 UI)
**Colorectal**						
Insufficient whole grains (<125 g/d)	26377 (23326-29216)	19.4 (17.1-21.4)	26268 (23241-29096)	19.3 (17.0-21.3)	114 (62-174)	0.08 (0.05-0.13)
Insufficient total dairy products (<3 serving/d)	17962 (16317-19572)	13.2 (12.0-14.4)	17962 (16317-19572)	13.2 (12.0-14.3)	0	0
High processed meat (>0 g/d)	11517 (9540-13727)	8.45 (7.01-10.05)	11401 (9404-13608)	8.35 (6.92-9.95)	123 (62-198)	0.09 (0.05-0.14)
High red meat (>14.3 g/d)	4591 (3080-6254)	3.37 (2.26-4.56)	4511 (2983-6165)	3.31 (2.19-4.50)	81 (43-136)	0.06 (0.03-0.10)
Insufficient fruits (<300 g/d)	228 (114-362)	0.17 (0.08-0.27)	0	0	228 (114-362)	0.17 (0.08-0.27)
High SSBs (>0 serving/d)	223 (138-332)	0.16 (0.10-0.24)	0	0	223 (138-332)	0.16 (0.10-0.24)
Insufficient vegetables (<400 g/d)	155 (88-261)	0.11 (0.06-0.19)	0	0	155 (88-261)	0.11 (0.06-0.19)
**Suboptimal diet**	39512 (36378-42686)	29.0 (26.6-31.3)	38845 (35619-41980)	28.5 (26.1-30.7)	936 (731-1152)	0.69 (0.54-0.85)
**Mouth, pharynx, larynx**						
Insufficient vegetables (<400 g/d)	10532 (8902-12060)	18.9 (16.0-21.7)	10532 (8902-12060)	18.9 (16.0-21.7)	0	0
Insufficient fruits (<300 g/d)	4787 (3632-6053)	8.59 (6.50-10.82)	4787 (3632-6053)	8.59 (6.50-10.82)	0	0
**Suboptimal diet**	14421 (12492-16146)	25.9 (22.6-28.9)	14421 (12492-16146)	62.9 (55.0-70.8)	0	0
**Corpus uteri**						
High SSBs (>0 serving/d)	832 (716-963)	1.60 (1.38-1.85)	0	0	832 (716-963)	1.60 (1.38-1.85)
Insufficient fruits (<300 g/d)	770 (649-899)	1.48 (1.25-1.73)	0	0	770 (649-899)	1.48 (1.25-1.73)
Insufficient vegetables (<400 g/d)	510 (404-615)	0.98 (0.78-1.18)	0	0	510 (404-615)	0.98 (0.78-1.18)
High processed meat (>0 g/d)	467 (405-537)	0.90 (0.78-1.03)	0	0	467 (405-537)	0.90 (0.78-1.03)
Insufficient whole grains (<125 g/d)	355 (316-389)	0.68 (0.61-0.75)	0	0	355 (316-389)	0.68 (0.61-0.75)
High red meat (>14.3 g/d)	324 (275-369)	0.62 (0.53-0.71)	0	0	324 (275-369)	0.62 (0.53-0.71)
**Suboptimal diet**	3180 (2965-3421)	6.11 (5.70-6.56)	0	0	3180 (2965-3421)	6.11 (5.70-6.56)
**Breast (post-menopausal)**						
High SSBs (>0 g/d)	738 (607-899)	0.38 (0.31-0.46)	0	0	738 (607-899)	0.38 (0.31-0.46)
Insufficient fruits (<300 g/d)	730 (590-904)	0.37 (0.30-0.46)	0	0	730 (590-904)	0.37 (0.30-0.46)
Insufficient vegetables (<400 g/d)	490 (373-629)	0.25 (0.19-0.32)	0	0	490 (373-629)	0.25 (0.19-0.32)
High processed meat (>0 g/d)	462 (383-563)	0.24 (0.20-0.29)	0	0	462 (383-563)	0.24 (0.20-0.29)
Insufficient whole grains (<125 g/d)	343 (287-401)	0.18 (0.15-0.20)	0	0	343 (287-401)	0.18 (0.15-0.20)
High red meat (>14.3 g/d)	310 (250-377)	0.16 (0.13-0.19)	0	0	310 (250-377)	0.16 (0.13-0.19)
**Suboptimal diet**	3074 (2799-3351)	1.57 (1.43-1.71)	0	0	3074 (2799-3351)	1.57 (1.43-1.71)
**Kidney**						
Insufficient fruits (<300 g/d)	518 (458-587)	0.87 (0.77-0.98)	0	0	518 (458-587)	0.87 (0.77-0.98)
High SSBs (>0 g/d)	498 (448-552)	0.83 (0.75-0.92)	0	0	498 (448-552)	0.83 (0.75-0.92)
Insufficient vegetables (<400 g/d)	349 (290-411)	0.58 (0.49-0.69)	0	0	349 (290-411)	0.58 (0.49-0.69)
Insufficient whole grains (<125 g/d)	252 (229-274)	0.42 (0.38-0.46)	0	0	252 (229-274)	0.42 (0.38-0.46)
High processed meat (>0 g/d)	263 (237-292)	0.44 (0.40-0.49)	0	0	263 (237-292)	0.44 (0.40-0.49)
High red meat (>14.3 g/d)	174 (154-193)	0.29 (0.26-0.32)	0	0	174 (154-193)	0.29 (0.26-0.32)
**Suboptimal diet**	2026 (1915-2140)	3.39 (3.21-3.57)	0	0	2026 (1915-2140)	3.39 (3.21-3.57)
**Stomach**						
High processed meat (>0 g/d)	1389 (1008-1751)	6.08 (4.42-7.67)	1364 (983-1723)	5.97 (4.32-7.56)	26 (19-34)	0.11 (0.08-0.15)
Insufficient fruits (<300 g/d)	51 (37-68)	0.22 (0.16-0.30)	0	0	51 (37-68)	0.22 (0.16-0.30)

High SSBs (>0 g/d)	44 (33-57)	0.19 (0.14-0.25)	0	0	44 (33-57)	0.19 (0.14-0.25)
Insufficient vegetables (<400 g/d)	36 (24-51)	0.16 (0.10-0.23)	0	0	36 (24-51)	0.16 (0.10-0.23)
Insufficient whole grains (<125 g/d)	26 (18-34)	0.11 (0.08-0.15)	0	0	26 (18-34)	0.11 (0.08-0.15)
High red meat (>14.3 g/d)	17 (12-22)	0.07 (0.05-0.09)	0	0	17 (12-22)	0.07 (0.05-0.09)
**Suboptimal diet**	1564 (1179-1922)	6.8 (5.2-8.4)	1364 (983-1723)	6.0 (4.3-7.6)	198 (172-225)	0.86 (0.75-0.98)
**Liver**						
Insufficient fruits (<300 g/d)	257 (213-314)	0.85 (0.71-1.03)	0	0	257 (213-314)	0.85 (0.71-1.03)
High SSBs (>0 g/d)	242 (205-283)	0.80 (0.67-0.93)	0	0	242 (205-283)	0.80 (0.67-0.93)
Insufficient vegetables (<400 g/d)	175 (140-216)	0.58 (0.46-0.71)	0	0	175 (140-216)	0.58 (0.46-0.71)
Insufficient whole grains (<125 g/d)	129 (108-152)	0.42 (0.36-0.50)	0	0	129 (108-152)	0.42 (0.36-0.50)
High processed meat (>0 g/d)	129 (108-151)	0.43 (0.36-0.50)	0	0	129 (108-151)	0.43 (0.36-0.50)
High red meat (>14.3 g/d)	84 (71-99)	0.28 (0.23-0.33)	0	0	84 (71-99)	0.28 (0.23-0.33)
**Suboptimal diet**	1,004 (928-1,085)	3.31 (3.07-3.59)	0	0	1,004 (928-1,085)	3.31 (3.07-3.59)
**Pancreas**						
Insufficient fruits (<300 g/d)	136 (109-163)	0.30 (0.24-0.36)	0	0	136 (109-163)	0.30 (0.24-0.36)
High SSBs (>0 g/d)	121 (103-144)	0.27 (0.23-0.32)	0	0	121 (103-144)	0.27 (0.23-0.32)
Insufficient vegetables (<400 g/d)	93 (75-116)	0.21 (0.17-0.26)	0	0	93 (75-116)	0.21 (0.17-0.26)
High processed meat (>0 g/d)	75 (62-90)	0.17 (0.14-0.20)	0	0	75 (62-90)	0.17 (0.14-0.20)
Insufficient whole grains (<125 g/d)	67 (56-79)	0.15 (0.12-0.18)	0	0	67 (56-79)	0.15 (0.12-0.18)
High red meat (>14.3 g/d)	48 (39-59)	0.11 (0.09-0.13)	0	0	48 (39-59)	0.11 (0.09-0.13)
**Suboptimal diet**	540 (494-586)	1.20 (1.10-1.30)	0	0	540 (494-586)	1.20 (1.10-1.30)
**Esophagus (adenocarcinoma)**						
Insufficient fruits (<300 g/d)	126 (101-156)	1.23 (0.98-1.51)	0	0	126 (101-156)	1.23 (0.98-1.51)
High SSBs (>0 g/d)	106 (86-127)	1.03 (0.83-1.23)	0	0	106 (86-127)	1.03 (0.83-1.23)
Insufficient vegetables (<400 g/d)	88 (66-114)	0.86 (0.65-1.10)	0	0	88 (66-114)	0.86 (0.65-1.10)
Insufficient whole grains (<125 g/d)	64 (54-76)	0.63 (0.53-0.74)	0	0	64 (54-76)	0.63 (0.53-0.74)
High processed meat (>0 g/d)	62 (52-74)	0.61 (0.51-0.71)	0	0	62 (52-74)	0.61 (0.51-0.71)
High red meat (>14.3 g/d)	40 (33-48)	0.38 (0.32-0.47)	0	0	40 (33-48)	0.38 (0.32-0.47)
**Suboptimal diet**	477 (433-529)	4.64 (4.24-5.09)	0	0	477 (433-529)	4.64 (4.24-5.09)
**Thyroid**						
High SSBs (>0 g/d)	118 (92-149)	0.25 (0.20-0.31)	0	0	118 (92-149)	0.25 (0.20-0.31)
Insufficient fruits (<300 g/d)	100 (79-124)	0.21 (0.17-0.26)	0	0	100 (79-124)	0.21 (0.17-0.26)
Insufficient vegetables (<400 g/d)	64 (50-80)	0.14 (0.11-0.17)	0	0	64 (50-80)	0.14 (0.11-0.17)
High processed meat (>0 g/d)	52 (40-64)	0.11 (0.09-0.13)	0	0	52 (40-64)	0.11 (0.09-0.13)
Insufficient whole grains (<125 g/d)	46 (37-54)	0.10 (0.08-0.11)	0	0	46 (37-54)	0.10 (0.08-0.11)
High red meat (>14.3 g/d)	37 (29-46)	0.08 (0.06-0.10)	0	0	37 (29-46)	0.08 (0.06-0.10)
**Suboptimal diet**	417 (376-463)	0.88 (0.80-0.98)	0	0	417 (376-463)	0.88 (0.80-0.98)
**Prostate (advanced)**						
Insufficient fruits (<300 g/d)	72 (37-112)	0.24 (0.12-0.38)	0	0	72 (37-112)	0.24 (0.12-0.38)
High SSBs (>0 g/d)	60 (33-90)	0.20 (0.11-0.30)	0	0	60 (33-90)	0.20 (0.11-0.30)
Insufficient vegetables (<400 g/d)	50 (25-81)	0.17 (0.08-0.27)	0	0	50 (25-81)	0.17 (0.08-0.27)
Insufficient whole grains (<125 g/d)	37 (20-57)	0.12 (0.07-0.19)	0	0	37 (20-57)	0.12 (0.07-0.19)
High processed meat (>0 g/d)	34 (17-52)	0.11 (0.06-0.17)	0	0	34 (17-52)	0.11 (0.06-0.17)
High red meat (>14.3 g/d)	20 (11-32)	0.07 (0.04-0.11)	0	0	20 (11-32)	0.07 (0.04-0.11)
**Suboptimal diet**	275 (215-336)	0.93 (0.73-1.14)	0	0	275 (215-336)	0.93 (0.73-1.14)
**Multiple myeloma**						

Insufficient fruits (<300 g/d)	61 (47-79)	0.28 (0.21-0.36)	0	0	61 (47-79)	0.28 (0.21-0.36)
High SSBs (>0 g/d)	57 (44-70)	0.26 (0.20-0.32)	0	0	57 (44-70)	0.26 (0.20-0.32)
Insufficient vegetables (<400 g/d)	41 (30-54)	0.19 (0.14-0.25)	0	0	41 (30-54)	0.19 (0.14-0.25)
High processed meat (>0 g/d)	33 (24-42)	0.15 (0.11-0.19)	0	0	33 (24-42)	0.15 (0.11-0.19)
Insufficient whole grains (<125 g/d)	30 (22-38)	0.14 (0.10-0.17)	0	0	30 (22-38)	0.14 (0.10-0.17)
High red meat (>14.3 g/d)	21 (15-28)	0.10 (0.07-0.12)	0	0	21 (15-28)	0.10 (0.07-0.12)
**Suboptimal diet**	241 (215-272)	1.10 (0.99-1.23)	0	0	241 (215-272)	1.10 (0.99-1.23)
**Ovary**						
High SSBs (>0 g/d)	44 (31-60)	0.21 (0.15-0.29)	0	0	44 (31-60)	0.21 (0.15-0.29)
Insufficient fruits (<300 g/d)	41 (27-57)	0.20 (0.13-0.28)	0	0	41 (27-57)	0.20 (0.13-0.28)
Insufficient vegetables (<400 g/d)	27 (17-39)	0.13 (0.08-0.19)	0	0	27 (17-39)	0.13 (0.08-0.19)
High processed meat (>0 g/d)	25 (16-35)	0.12 (0.08-0.17)	0	0	25 (16-35)	0.12 (0.08-0.17)
Insufficient whole grains (<125 g/d)	19 (13-26)	0.09 (0.06-0.12)	0	0	19 (13-26)	0.09 (0.06-0.12)
High red meat (>14.3 g/d)	17 (11-24)	0.08 (0.05-0.12)	0	0	17 (11-24)	0.08 (0.05-0.12)
Suboptimal diet	174 (147-200)	0.84 (0.71-0.97)	0	0	174 (147-200)	0.84 (0.71-0.97)
**Gallbladder**						
Insufficient fruits (<300 g/d)	26 (21-32)	0.69 (0.56-0.84)	0	0	26 (21-32)	0.69 (0.56-0.84)
High SSBs (>0 serving/d)	24 (20-30)	0.65 (0.54-0.78)	0	0	24 (20-30)	0.65 (0.54-0.78)
High processed meat (>0 g/d)	15 (12-19)	0.41 (0.33-0.50)	0	0	15 (12-19)	0.41 (0.33-0.50)
Insufficient vegetables (<400 g/d)	18 (14-23)	0.48 (0.38-0.60)	0	0	18 (14-23)	0.48 (0.38-0.60)
Insufficient whole grains (<125 g/d)	13 (11-15)	0.34 (0.29-0.40)	0	0	13 (11-15)	0.34 (0.29-0.40)
High red meat (>14.3 g/d)	10 (8-13)	0.27 (0.22-0.33)	0	0	10 (8-13)	0.27 (0.22-0.33)
**Suboptimal diet**	106 (96-117)	2.82 (2.60-3.09)	0	0	106 (96-117)	2.82 (2.60-3.09)

PAF: Population Attributable Fraction; UI: Uncertainty Intervals

Table 5. Estimated Number and Proportion of New Cancer Cases Associated with Suboptimal Dietary Intake Among US Adults in 2015, by Cancer Site and Population Subgroups

Cancer type	Burden	20-44 y		45-54 y		55-64 y		65+ y		Overall
		Men	Women	Men	Women	Men	Women	Men	Women	
**Colorectal**										
Age	N	3287 (2995-3593)		7723 (7047-8332)		12006 (10916-13073)		29277 (26451-31901)		52225 (49264-55302)
	PAF, %	38.5 (35.4-41.5)		38.6 (35.3-41.7)		38.6 (35.2-41.9)		38.1 (34.6-41.6)		38.3 (36.1-40.4)
Race/ethnicity										
White	N	1052 (856-1256)	1041 (845-1225)	2789 (2330-3236)	2355 (1940-2774)	4699 (3881-5540)	3617 (2959-4185)	11040 (8993-12833)	12076 (9888-13948)	38545 (35424-41594)
	PAF, %	36.8 (30.2-42.7)	40.1 (32.4-46.2)	36.7 (30.7-42.5)	40.2 (33.1-46.9)	37.0 (30.6-43.3)	40.1 (32.8-46.0)	36.5 (30.0-42.7)	39.6 (32.7-45.4)	38.1 (35.0-40.9)
Black	N	224 (171-282)	249 (188-317)	604 (497-711)	639 (536-752)	1010 (835-1188)	931 (763-1086)	1438 (1195-1680)	1621 (1344-1904)	6722 (6238-7201)
	PAF, %	38.7 (32.2-45.1)	42.1 (34.7-47.9)	39.1 (32.4-44.7)	42.4 (35.3-48.8)	39.2 (32.2-45.5)	42.5 (35.3-49.0)	38.7 (32.4-44.4)	40.8 (34.0-46.9)	40.1 (37.7-42.6)
Hispanic	N	255 (193-317)	254 (197-321)	453 (367-546)	436 (350-519)	644 (523-767)	507 (411-610)	1073 (848-1280)	990 (803-1173)	4610 (4260-4978)
	PAF, %	36.8 (30.1-42.9)	39.8 (32.4-46.3)	36.6 (30.4-42.4)	40.2 (33.3-46.3)	36.9 (30.3-43.1)	39.8 (32.4-46.1)	36.5 (29.8-42.4)	39.5 (33.0-45.6)	38.0 (35.5-40.4)
Other	N	97 (68-128)	114 (76-160)	215 (166-268)	218 (168-272)	337 (266-408)	271 (209-333)	553 (421-702)	570 (436-709)	2377 (2167-2621)
	PAF, %	37.5 (31.1-43.3)	40.6 (33.8-47.0)	37.4 (31.0-43.2)	40.2 (33.5-46.3)	37.9 (31.3-43.5)	40.5 (33.7-46.8)	37.1 (30.6-43.4)	39.4 (32.2-45.2)	38.5 (35.7-40.9)
Total	N	1629 (1417-1857)	1663 (1433-1881)	4066 (3585-4511)	3657 (3201-4085)	6695 (5867-7535)	5320 (4648-5964)	14118 (12020-15968)	15243 (13096-17179)	
	PAF, %	37.0 (32.8-41.0)	40.2 (35.2-44.6)	37.1 (32.7-41.1)	40.4 (35.6-45.0)	37.3 (32.7-42.0)	40.5 (35.2-44.7)	36.7 (31.4-41.6)	39.6 (34.2-44.5)	
**Mouth, pharynx and larynx**										
Age	N	767 (621-913)		2396 (1869-2830)		4768 (3722-5647)		6525 (5103-7918)		14421 (12492-16146)
	PAF, %	27.8 (22.7-32.6)		26.6 (20.9-31.5)		26.5 (20.6-31.4)		25.2 (19.6-30.3)		25.9 (22.6-28.9)
Race/ethnicity										
White	N	303 (188-414)	194 (118-272)	1379 (909-1798)	473 (297-638)	2960 (1955-3810)	892 (598-1177)	3811 (2555-4990)	1646 (1059-2163)	11595 (9756-13272)
	PAF, %	27.1 (17.3-36.4)	29.2 (18.7-39.0)	26.4 (17.3-34.1)	27.6 (17.7-37.1)	26.5 (17.5-34.2)	27.0 (18.1-34.9)	24.8 (16.5-32.8)	26.8 (17.2-35.1)	25.9 (21.8-29.6)
Black	N	58 (32-89)	38 (20-61)	185 (128-238)	79 (51-109)	393 (273-505)	148 (102-190)	373 (264-484)	146 (97-202)	1422 (1235-1608)
	PAF, %	29.7 (18.4-39.5)	30.4 (19.7-40.7)	28.3 (20.1-36.1)	29.4 (19.6-38.4)	27.2 (18.7-34.3)	29.1 (20.6-36.6)	25.4 (18.6-32.1)	27.8 (19.0-35.8)	27.3 (23.8-30.6)
Hispanic	N	53 (32-80)	46 (24-72)	120 (78-160)	47 (29-65)	188 (129-249)	61 (39-83)	256 (178-345)	111 (66-159)	882 (759-1010)
	PAF, %	26.6 (17.7-35.2)	27.6 (16.8-38.3)	24.0 (16.1-31.5)	26.1 (16.8-33.4)	24.7 (16.7-31.7)	26.1 (17.2-33.8)	23.9 (17.0-30.5)	25.1 (15.9-32.8)	24.7 (21.5-27.9)
Other	N	47 (25-69)	25 (12-40)	73 (44-105)	41 (25-58)	87 (55-127)	50 (31-69)	117 (75-165)	74 (45-107)	516 (438-605)
	PAF, %	26.4 (15.6-35.0)	27.6 (16.8-37.4)	25.5 (16.1-33.9)	26.5 (17.1-34.3)	23.7 (15.1-31.4)	25.9 (16.8-33.8)	22.7 (15.5-29.7)	23.9 (14.6-31.4)	24.4 (21.3-27.4)
		464 (349-583)	303 (218-392)	1756 (1259-2173)	637 (466-795)	3613 (2620-4476)	1150 (850-1444)	4568 (3262-5775)	1977 (1387-2490)	
		27.2 (20.7-33.7)	29.0 (21.5-35.9)	26.3 (19.0-32.4)	27.6 (20.1-34.4)	26.3 (18.9-32.4)	27.1 (19.8-33.6)	24.7 (17.9-31.4)	26.6 (18.5-33.6)	
**Corpus uteri**										
Age	N	255 (225-288)		562 (490-636)		1205 (1056-1367)		1158 (1034-1290)		3180 (2965-3421)
	PAF, %	7.3 (6.6-8.1)		7.1 (6.3-8.1)		6.7 (5.9-7.6)		5.1 (4.6-5.7)		6.1 (5.7-6.6)
Race/ethnicity										
White	N	0	142 (117-168)	0	390 (323-460)	0	895 (753-1048)	0	910 (788-1039)	2337 (2123-2571)
	PAF, %	0	7.1 (6.0-8.2)	0	7.1 (5.9-8.4)	0	6.6 (5.5-7.7)	0	5.1 (4.4-5.8)	6.0 (5.5-6.6)
Black	N	0	31 (23-39)	0	58 (46-71)	0	159 (129-189)	0	139 (117-162)	387 (346-428)
	PAF, %	0	7.8 (6.4-9.1)	0	7.8 (6.4-9.3)	0	7.5 (6.1-8.8)	0	5.4 (4.6-6.1)	6.6 (6.0-7.3)
Hispanic	N	0	63 (49-79)	0	77 (63-93)	0	107 (88-131)	0	73 (61-88)	321 (289-357)
	PAF, %	0	8.0 (6.6-9.4)	0	7.1 (5.8-8.4)	0	6.8 (5.8-8.1)	0	5.1 (4.4-5.8)	6.6 (6.0-7.2)
Other	N	0	20 (13-27)	0	34 (27-43)	0	43 (35-55)	0	35 (27-45)	134 (117-152)
	PAF, %	0	6.7 (5.6-7.8)	0	6.4 (5.2-7.5)	0	5.7 (4.8-6.8)	0	5.1 (4.3-5.9)	5.8 (5.3-6.3)
Total	N	0	255 (225-288)	0	562 (490-636)	0	1205 (1056-1367)	0	1158 (1034-1290)	
	PAF, %	0	7.3 (6.6-8.1)	0	7.1 (6.3-8.1)	0	6.7 (5.9-7.6)	0	5.1 (4.6-5.7)	

Cancer type	Burden	20-44 y		45-54 y		55-64 y		65+ y		Overall
		Men	Women	Men	Women	Men	Women	Men	Women	
**Breast (post-menopausal)**										
Age	N	0		484 (416-557)		1105 (943-1275)		1476 (1270-1688)		3074 (2799-3351)
	PAF, %	0		1.9 (1.7-2.2)		1.8 (1.5-2.1)		1.4 (1.2-1.6)		1.6 (1.4-1.7)
Race/ethnicity										
White	N	0	0	0	340 (275-410)	0	814 (655-974)	0	1199 (990-1411)	2356 (2084-2631)
	PAF, %	0	1.9 (1.5-2.3)	0	1.9 (1.6-2.3)	0	1.8 (1.4-2.1)	0	1.4 (1.1-1.6)	1.5 (1.4-1.7)
Black	N	0	0	0	73 (58-90)	0	157 (124-193)	0	146 (121-177)	377 (335-427)
	PAF, %	0	2.1 (1.7-2.5)	0	2.1 (1.7-2.6)	0	2.0 (1.6-2.5)	0	1.4 (1.2-1.7)	1.8 (1.6-2.0)
Hispanic	N	0	0	0	47 (37-59)	0	89 (71-110)	0	84 (69-101)	221 (195-248)
	PAF, %	0	2.1 (1.7-2.6)	0	1.9 (1.5-2.3)	0	1.8 (1.5-2.2)	0	1.4 (1.1-1.6)	1.6 (1.4-1.8)
Other	N	0	0	0	24 (19-30)	0	46 (37-56)	0	48 (38-60)	118 (103-134)
	PAF, %	0	1.8 (1.5-2.2)	0	1.7 (1.4-2.1)	0	1.5 (1.2-1.9)	0	1.4 (1.1-1.6)	1.5 (1.3-1.7)
Total	N	0	0	0	484 (416-557)	0	1105 (943-1275)	0	1476 (1270-1688)	
	PAF, %	0	0	0	1.9 (1.7-2.2)	0	1.8 (1.5-2.1)	0	1.4 (1.2-1.6)	
**Kidney**										
Age	N	200 (180-223)		351 (318-383)		607 (546-672)		869 (792-950)		2026 (1915-2140)
	PAF, %	4.2 (3.8-4.5)		3.9 (3.5-4.2)		3.8 (3.4-4.1)		2.9 (2.7-3.2)		3.4 (3.2-3.6)
Race/ethnicity										
White	N	69 (55-83)	51 (41-63)	144 (118-171)	88 (72-106)	271 (221-326)	159 (132-190)	409 (344-479)	272 (233-318)	1465 (1355-1573)
	PAF, %	3.7 (3.1-4.4)	4.4 (3.6-5.2)	3.5 (2.9-4.1)	4.4 (3.6-5.2)	3.5 (2.9-4.2)	4.0 (3.4-4.8)	2.8 (2.3-3.2)	3.1 (2.7-3.6)	3.3 (3.1-3.5)
Black	N	19 (14-25)	13 (9-17)	33 (26-40)	22 (18-28)	59 (47-72)	37 (30-45)	50 (40-60)	42 (35-51)	276 (255-301)
	PAF, %	4.7 (3.9-5.7)	4.8 (4.0-5.8)	4.0 (3.2-4.7)	4.9 (4.0-5.8)	4.0 (3.2-4.8)	4.6 (3.8-5.5)	3.0 (2.5-3.5)	3.3 (2.8-3.8)	3.8 (3.6-4.1)
Hispanic	N	19 (15-25)	19 (14-25)	28 (23-35)	22 (18-28)	37 (29-45)	28 (22-34)	40 (32-48)	30 (24-37)	225 (207-244)
	PAF, %	4.1 (3.4-4.9)	4.9 (4.0-5.8)	3.5 (2.9-4.1)	4.3 (3.6-5.1)	3.5 (2.8-4.1)	4.2 (3.5-5.0)	2.8 (2.4-3.3)	3.1 (2.6-3.6)	3.6 (3.3-3.8)
Other	N	5 (2-9)	3 (2-5)	8 (5-11)	4 (2-7)	10 (7-13)	6 (4-9)	14 (10-18)	11 (7-15)	62 (53-70)
	PAF, %	3.6 (2.9-4.3)	4.1 (3.4-4.9)	3.2 (2.6-3.8)	3.9 (3.1-4.6)	3.2 (2.6-3.9)	3.5 (2.9-4.2)	2.4 (2.0-2.8)	3.1 (2.6-3.6)	3.1 (2.9-3.3)
Total	N	113 (97-130)	87 (75-101)	213 (185-242)	138 (120-156)	376 (325-431)	230 (199-262)	513 (447-585)	356 (314-403)	
	PAF, %	3.9 (3.5-4.4)	4.5 (4.0-5.0)	3.6 (3.1-4.0)	4.4 (3.9-5.0)	3.6 (3.1-4.0)	4.1 (3.6-4.7)	2.8 (2.4-3.1)	3.1 (2.8-3.5)	
**Stomach**										
Age	N	97 (64-129)		181 (125-242)		349 (236-462)		937 (577-1265)		1564 (1179-1922)
	PAF, %	7.9 (5.1-11.0)		6.9 (4.8-9.3)		6.7 (4.5-8.9)		6.8 (4.1-9.3)		6.8 (5.2-8.4)
Race/ethnicity										
White	N	17 (4-29)	22 (3-46)	42 (16-67)	43 (5-76)	105 (40-167)	91 (13-164)	298 (107-496)	279 (36-503)	894 (537-1220)
	PAF, %	5.7 (1.4-11.1)	9.7 (1.1-22.9)	4.8 (1.9-7.6)	8.7 (1.1-15.9)	4.8 (1.9-7.7)	9.0 (1.3-16.4)	4.9 (1.8-8.2)	8.7 (1.1-16.0)	6.2 (3.7-8.5)
Black	N	7 (2-15)	12 (1-25)	19 (2-36)	23 (3-45)	43 (8-79)	31 (7-58)	79 (5-147)	87 (12-158)	302 (186-424)
	PAF, %	7.4 (1.7-18.7)	10.3 (0.7-23.7)	7.3 (0.8-13.8)	10.5 (1.3-21.9)	7.0 (1.2-13.0)	10.1 (2.1-19.6)	7.5 (0.5-14.2)	10.2 (1.5-19.6)	8.7 (5.3-12.1)
Hispanic	N	10 (2-20)	17 (2-33)	17 (3-33)	21 (3-38)	24 (4-46)	25 (3-48)	53 (7-101)	59 (6-110)	226 (146-310)
	PAF, %	5.8 (1.1-12.7)	8.9 (0.8-19.0)	5.6 (1.1-11.1)	8.7 (1.3-16.2)	5.9 (1.0-11.3)	8.5 (1.2-16.8)	5.8 (0.7-11.4)	8.9 (0.8-17.0)	7.1 (4.6-9.7)
Other	N	3 (1-8)	6 (0-14)	7 (1-13)	10 (1-19)	15 (3-28)	15 (1-30)	37 (5-73)	42 (2-84)	136 (84-196)
	PAF, %	5.9 (1.1-16.0)	9.2 (0.7-25.4)	5.7 (1.1-11.4)	9.4 (1.4-18.8)	6.3 (1.2-12.6)	10.0 (0.7-20.5)	6.0 (1.0-11.9)	9.6 (0.5-20.2)	7.6 (4.6-11.1)
Total	N	38 (22-55)	59 (30-88)	85 (52-124)	96 (52-144)	187 (111-263)	162 (81-247)	470 (246-680)	465 (212-715)	
	PAF, %	6.1 (3.4-8.9)	9.6 (4.8-15.3)	5.4 (3.2-7.9)	9.2 (5.0-13.9)	5.4 (3.2-7.6)	9.2 (4.6-14.0)	5.4 (2.8-7.9)	9.0 (4.1-13.8)	
**Liver**										
Age	N	32 (26-38)		120 (105-137)		417 (364-481)		436 (381-490)		1004 (928-1085)
	PAF, %	4.2 (3.8-4.7)		3.7 (3.3-4.2)		3.7 (3.2-4.2)		2.9 (2.5-3.3)		3.3 (3.1-3.6)
Race/ethnicity										
White	N	7 (4-11)	7 (5-10)	46 (33-59)	19 (15-26)	190 (141-244)	57 (44-72)	184 (140-235)	100 (77-126)	613 (544-692)

Cancer type	Burden	20-44 y		45-54 y		55-64 y		65+ y		Overall
		Men	Women	Men	Women	Men	Women	Men	Women	
	PAF, %	3.7 (2.8-4.6)	4.4 (3.3-5.5)	3.5 (2.6-4.5)	4.4 (3.3-5.6)	3.5 (2.6-4.5)	4.0 (3.1-5.0)	2.7 (2.1-3.5)	3.1 (2.5-3.9)	3.2 (2.9-3.7)
Black	N	4 (2-8)	3 (1-4)	13 (10-17)	7 (5-10)	64 (47-84)	21 (16-28)	35 (26-46)	17 (13-23)	166 (143-190)
	PAF, %	4.7 (3.4-6.0)	4.7 (3.6-6.2)	3.9 (2.9-5.0)	4.9 (3.6-6.2)	4.0 (3.0-5.1)	4.6 (3.5-5.9)	3.0 (2.3-3.8)	3.3 (2.6-4.2)	3.8 (3.3-4.2)
Hispanic	N	4 (2-7)	3 (1-4)	19 (13-25)	5 (3-7)	44 (33-59)	16 (11-21)	35 (26-46)	25 (18-32)	152 (133-171)
	PAF, %	4.1 (3.0-5.3)	4.9 (3.6-6.3)	3.5 (2.6-4.5)	4.3 (3.3-5.4)	3.4 (2.6-4.5)	4.2 (3.1-5.4)	2.8 (2.1-3.5)	3.1 (2.4-3.9)	3.3 (3.0-3.7)
Other	N	3 (1-4)	1 (1-1)	8 (6-11)	2 (1-3)	19 (13-25)	6 (4-8)	20 (15-27)	15 (10-21)	73 (63-84)
	PAF, %	3.6 (2.7-4.6)	4.1 (3.1-5.3)	3.1 (2.4-4.1)	3.9 (2.9-5.0)	3.2 (2.4-4.2)	3.5 (2.7-4.5)	2.4 (1.9-3.0)	3.1 (2.4-4.0)	3.0 (2.7-3.4)
Total	N	19 (14-24)	13 (10-16)	86 (73-101)	34 (28-41)	317 (265-376)	100 (85-117)	276 (228-327)	158 (132-186)	
	PAF, %	4.0 (3.4-4.6)	4.5 (3.8-5.3)	3.5 (3.0-4.1)	4.4 (3.8-5.2)	3.5 (3.0-4.2)	4.1 (3.5-4.8)	2.8 (2.3-3.2)	3.1 (2.7-3.7)	
**Esophagus (adenocarcinoma)**										
Age	N	12 (9-16)		58 (49-67)		150 (126-176)		259 (224-297)		477 (433-529)
	PAF, %	5.8 (5.1-6.5)		5.4 (4.7-6.2)		5.3 (4.5-6.2)		4.2 (3.6-4.7)		4.6 (4.2-5.1)
Race/ethnicity										
White	N	7 (4-11)	1 (1-1)	40 (31-48)	7 (5-10)	113 (90-139)	17 (13-22)	193 (158-230)	39 (30-49)	415 (373-466)
	PAF, %	5.5 (4.4-6.5)	6.5 (5.3-7.9)	5.2 (4.2-6.2)	6.5 (5.3-7.8)	5.2 (4.2-6.3)	5.9 (4.9-7.1)	4.1 (3.4-4.8)	4.6 (3.9-5.4)	4.6 (4.1-5.1)
Black	N	1 (1-2)	0 (0-0)	2 (1-3)	1 (1-2)	4 (3-6)	2 (2-3)	5 (3-7)	4 (3-5)	20 (17-23)
	PAF, %	6.9 (5.4-8.6)	7.1 (5.8-8.5)	5.9 (4.7-7.2)	7.2 (5.6-8.7)	5.9 (4.7-7.1)	6.8 (5.5-8.2)	4.4 (3.7-5.2)	4.9 (4.2-5.8)	5.5 (5.0-5.9)
Hispanic	N	2 (1-3)	0 (0-0)	4 (2-5)	1 (1-2)	7 (5-10)	2 (2-3)	9 (6-13)	3 (3-4)	29 (25-34)
	PAF, %	6.1 (4.9-7.4)	7.2 (5.8-8.7)	5.1 (4.2-6.3)	6.4 (5.2-7.7)	5.1 (4.2-6.2)	6.3 (5.1-7.6)	4.2 (3.5-5.0)	4.6 (3.9-5.4)	4.9 (4.5-5.3)
Other	N	1 (0-1)	0 (0-0)	2 (1-2)	1 (0-1)	2 (2-3)	1 (1-1)	4 (3-5)	2 (2-3)	13 (11-14)
	PAF, %	5.4 (4.3-6.5)	6.1 (5.0-7.3)	4.7 (3.9-5.7)	5.7 (4.6-6.9)	4.7 (3.8-5.8)	5.2 (4.3-6.4)	3.6 (3.0-4.3)	4.6 (3.8-5.4)	4.4 (4.0-4.8)
Total	N	11 (7-15)	1 (1-2)	47 (39-56)	10 (8-13)	127 (104-153)	22 (18-27)	211 (176-247)	48 (39-58)	
	PAF, %	5.7 (5.0-6.5)	6.6 (5.9-7.5)	5.2 (4.4-6.1)	6.5 (5.6-7.5)	5.2 (4.3-6.2)	6.0 (5.2-6.9)	4.1 (3.5-4.7)	4.6 (4.0-5.3)	
**Pancreas**										
Age	N	15 (13-18)		56 (49-64)		146 (127-166)		322 (283-363)		540 (494-586)
	PAF, %	1.5 (1.4-1.7)		1.5 (1.3-1.6)		1.4 (1.2-1.6)		1.1 (1.0-1.2)		1.2 (1.1-1.3)
Race/ethnicity										
White	N	5 (4-7)	5 (3-7)	19 (15-25)	18 (14-23)	55 (41-70)	49 (38-61)	121 (96-150)	130 (105-162)	404 (359-450)
	PAF, %	1.3 (1.0-1.7)	1.6 (1.3-2.0)	1.3 (1.0-1.6)	1.6 (1.2-2.0)	1.3 (1.0-1.6)	1.5 (1.2-1.9)	1.0 (0.8-1.2)	1.1 (0.9-1.4)	1.2 (1.0-1.3)
Black	N	1 (0-2)	1 (1-2)	5 (4-7)	6 (4-7)	12 (9-16)	14 (10-17)	14 (11-18)	23 (17-28)	76 (68-85)
	PAF, %	1.7 (1.3-2.2)	1.8 (1.3-2.2)	1.5 (1.1-1.9)	1.8 (1.4-2.3)	1.5 (1.1-1.9)	1.7 (1.3-2.1)	1.1 (0.9-1.4)	1.2 (1.0-1.5)	1.4 (1.2-1.5)
Hispanic	N	1 (0-2)	1 (1-2)	3 (2-4)	3 (2-4)	6 (4-8)	5 (4-7)	10 (8-13)	12 (9-15)	42 (37-47)
	PAF, %	1.5 (1.2-1.9)	1.8 (1.4-2.3)	1.3 (1.0-1.6)	1.6 (1.2-2.0)	1.3 (1.0-1.6)	1.5 (1.2-2.0)	1.0 (0.8-1.3)	1.1 (0.9-1.4)	1.2 (1.1-1.4)
Other	N	0 (0-0)	0 (0-0)	1 (1-2)	1 (1-1)	2 (2-3)	2 (1-3)	5 (3-6)	7 (5-9)	18 (15-21)
	PAF, %	1.3 (1.0-1.7)	1.5 (1.2-1.9)	1.2 (0.9-1.5)	1.4 (1.1-1.8)	1.2 (0.9-1.5)	1.3 (1.0-1.6)	0.9 (0.7-1.1)	1.1 (0.9-1.4)	1.1 (1.0-1.2)
Total	N	7 (6-10)	8 (6-10)	29 (24-35)	27 (22-32)	76 (62-92)	69 (58-83)	150 (124-179)	171 (145-203)	
	PAF, %	1.4 (1.2-1.7)	1.7 (1.4-2.0)	1.3 (1.1-1.6)	1.6 (1.4-1.9)	1.3 (1.1-1.6)	1.5 (1.3-1.8)	1.0 (0.8-1.2)	1.1 (1.0-1.4)	
**Thyroid**										
Age	N	159 (131-192)		97 (80-118)		90 (74-109)		68 (56-82)		417 (376-463)
	PAF, %	1.0 (0.8-1.2)		0.9 (0.8-1.1)		0.9 (0.7-1.1)		0.7 (0.6-0.8)		0.9 (0.8-1.0)
Race/ethnicity										
White	N	18 (12-23)	87 (62-117)	15 (10-20)	52 (35-72)	18 (12-25)	47 (32-64)	18 (12-24)	36 (25-46)	292 (253-335)
	PAF, %	0.8 (0.5-1.1)	1.0 (0.7-1.3)	0.8 (0.5-1.1)	1.0 (0.7-1.3)	0.8 (0.5-1.1)	0.9 (0.6-1.2)	0.6 (0.4-0.8)	0.7 (0.5-0.9)	0.9 (0.7-1.0)
Black	N	2 (1-3)	11 (7-15)	1 (1-2)	8 (5-11)	2 (1-2)	7 (5-10)	1 (1-2)	4 (3-6)	37 (32-43)
	PAF, %	1.1 (0.7-1.5)	1.1 (0.7-1.5)	0.9 (0.6-1.2)	1.1 (0.7-1.5)	0.9 (0.6-1.2)	1.0 (0.7-1.4)	0.7 (0.5-0.9)	0.7 (0.5-1.0)	1.0 (0.8-1.1)
Hispanic	N	4 (3-6)	25 (16-36)	2 (1-3)	12 (8-17)	2 (1-3)	8 (6-11)	2 (1-2)	4 (3-6)	59 (49-71)
	PAF, %	0.9 (0.6-1.3)	1.1 (0.7-1.6)	0.8 (0.5-1.1)	1.0 (0.7-1.3)	0.8 (0.5-1.1)	0.9 (0.6-1.3)	0.6 (0.4-0.8)	0.7 (0.5-0.9)	1.0 (0.8-1.1)

Cancer type	Burden	20-44 y		45-54 y		55-64 y		65+ y		Overall
		**Men**	**Women**	**Men**	**Women**	**Men**	**Women**	**Men**	**Women**	
Other	N	2 (1-3)	10 (7-14)	1 (1-2)	5 (3-7)	1 (1-2)	4 (3-6)	1 (1-1)	3 (2-4)	27 (23-33)
	PAF, %	0.8 (0.5-1.1)	0.9 (0.7-1.2)	0.7 (0.5-1.0)	0.9 (0.6-1.2)	0.7 (0.5-1.0)	0.8 (0.5-1.1)	0.5 (0.4-0.7)	0.7 (0.5-0.9)	0.8 (0.7-1.0)
Total	N	26 (19-32)	133 (106-166)	20 (15-26)	77 (61-98)	23 (17-30)	67 (52-84)	21 (16-28)	47 (36-58)	
	PAF, %	0.8 (0.6-1.0)	1.0 (0.8-1.2)	0.8 (0.6-1.0)	1.0 (0.8-1.2)	0.8 (0.6-1.0)	0.9 (0.7-1.1)	0.6 (0.5-0.8)	0.7 (0.5-0.9)	
**Prostate (advanced)**										
Age	N	2 (1 3)		32 (22 42)		115 (79 153)		172 (115 237)		322 (251 397)
	PAF, %	1.5 (1.1 2.0)		1.3 (0.9 1.7)		1.2 (0.8 1.6)		1.0 (0.7 1.4)		1.1 (0.8 1.3)
Race/ethnicity		1 (0 2)	0	19 (11 28)	0	76 (44 113)	0	134 (78 198)	0	233 (164 308)
White	N	1.4 (0.8 2.2)	0	1.2 (0.7 1.8)	0	1.1 (0.6 1.7)	0	1.0 (0.6 1.5)	0	1.1 (0.8 1.4)
	PAF, %	1 (0 1)	0	9 (5 14)	0	27 (15 41)	0	23 (14 35)	0	61 (44 79)
Black	N	1.7 (0.9 2.7)	0	1.4 (0.8 2.2)	0	1.4 (0.8 2.1)	0	1.0 (0.6 1.6)	0	1.3 (0.9 1.6)
	PAF, %	0 (0 0)	0	3 (2 4)	0	8 (5 13)	0	10 (6 15)	0	21 (15 28)
Hispanic	N	1.5 (0.8 2.4)	0	1.3 (0.7 1.9)	0	1.2 (0.7 1.8)	0	0.9 (0.5 1.3)	0	1.0 (0.8 1.4)
	PAF, %	0 (0 0)	0	1 (0 1)	0	2 (1 4)	0	4 (2 6)	0	7 (5 10)
Other	N	1.3 (0.7 2.0)	0	0.9 (0.5 1.4)	0	1.0 (0.5 1.6)	0	0.8 (0.4 1.1)	0	0.9 (0.6 1.1)
	PAF, %	2 (1 3)	0	32 (22 42)	0	115 (79 153)	0	172 (115 237)	0	
Total	N	1.5 (1.1 2.0)	0	1.3 (0.9 1.7)	0	1.2 (0.8 1.6)	0	1.0 (0.7 1.4)	0	
	PAF, %	1 (0 2)	0	19 (11 28)	0	76 (44 113)	0	134 (78 198)	0	233 (164 308)
**Multiple myeloma**										
Age	N	9 (7-11)		31 (26-36)		66 (55-79)		136 (114-163)		241 (215-272)
	PAF, %	1.4 (1.2-1.6)		1.3 (1.1-1.6)		1.3 (1.1-1.5)		1.0 (0.8-1.2)		1.1 (1.0-1.2)
Race/ethnicity										
White	N	2 (1-3)	2 (1-3)	8 (5-12)	7 (5-10)	22 (14-31)	18 (12-25)	52 (35-72)	44 (30-60)	155 (131-185)
	PAF, %	1.2 (0.8-1.7)	1.4 (0.9-2.0)	1.2 (0.8-1.6)	1.5 (1.0-2.0)	1.2 (0.7-1.6)	1.3 (0.9-1.9)	0.9 (0.6-1.2)	1.0 (0.7-1.4)	1.1 (0.9-1.3)
Black	N	2 (1-3)	1 (1-2)	4 (3-6)	5 (3-8)	9 (5-12)	9 (6-13)	12 (8-16)	14 (9-20)	56 (47-65)
	PAF, %	1.6 (1.0-2.2)	1.6 (1.0-2.2)	1.3 (0.9-1.9)	1.6 (1.1-2.3)	1.3 (0.9-1.8)	1.5 (0.9-2.1)	1.0 (0.7-1.4)	1.1 (0.7-1.5)	1.2 (1.0-1.4)
Hispanic	N	1 (0-2)	1 (0-1)	2 (1-3)	2 (1-3)	3 (2-5)	3 (2-4)	5 (3-7)	5 (3-8)	22 (18-26)
	PAF, %	1.4 (0.9-1.9)	1.6 (1.0-2.3)	1.2 (0.7-1.6)	1.5 (1.0-2.0)	1.1 (0.7-1.6)	1.4 (0.9-2.0)	0.9 (0.6-1.3)	1.0 (0.7-1.4)	1.1 (1.0-1.3)
Other	N	0 (0-0)	0 (0-0)	1 (0-1)	1 (0-1)	1 (1-2)	1 (1-1)	2 (1-3)	2 (1-3)	8 (6-9)
	PAF, %	1.2 (0.8-1.7)	1.4 (0.8-1.9)	1.0 (0.7-1.5)	1.3 (0.8-1.8)	1.1 (0.7-1.5)	1.2 (0.8-1.6)	0.8 (0.5-1.1)	1.0 (0.7-1.4)	1.0 (0.9-1.2)
Total	N	5 (3-6)	4 (3-5)	15 (12-19)	15 (12-20)	35 (26-45)	31 (23-39)	70 (54-91)	66 (51-82)	
	PAF, %	1.3 (1.1-1.7)	1.5 (1.2-1.9)	1.2 (0.9-1.5)	1.5 (1.2-1.8)	1.2 (0.9-1.5)	1.4 (1.1-1.8)	0.9 (0.7-1.2)	1.1 (0.8-1.3)	
**Ovary**										
Age	N	22 (16-29)		35 (26-45)		48 (34-64)		68 (49-88)		174 (147-200)
	PAF, %	1.0 (0.8-1.3)		1.0 (0.7-1.3)		0.9 (0.6-1.2)		0.7 (0.5-0.9)		0.8 (0.7-1.0)
Race/ethnicity										
White	N	0	13 (8-19)	0	24 (15-34)	0	36 (23-51)	0	55 (36-76)	129 (102-155)
	PAF, %	0	1.0 (0.6-1.4)	0	1.0 (0.6-1.4)	0	0.9 (0.6-1.3)	0	0.7 (0.5-0.9)	0.8 (0.7-1.0)
Black	N	0	2 (1-4)	0	4 (2-5)	0	5 (3-8)	0	6 (4-8)	17 (13-21)
	PAF, %	0	1.1 (0.7-1.6)	0	1.1 (0.7-1.6)	0	1.0 (0.6-1.5)	0	0.7 (0.5-1.1)	0.9 (0.7-1.1)
Hispanic	N	0	5 (3-7)	0	5 (3-7)	0	5 (3-7)	0	5 (3-7)	19 (15-23)
	PAF, %	0	1.1 (0.7-1.6)	0	1.0 (0.6-1.4)	0	1.0 (0.6-1.4)	0	0.7 (0.4-1.0)	0.9 (0.7-1.1)
Other	N	0	2 (1-3)	0	2 (1-3)	0	2 (1-4)	0	2 (1-3)	9 (7-11)
	PAF, %	0	0.9 (0.6-1.3)	0	0.9 (0.6-1.3)	0	0.8 (0.5-1.1)	0	0.7 (0.5-1.0)	0.8 (0.7-1.0)
Total	N	0	22 (16-29)	0	35 (26-45)	0	48 (34-64)	0	68 (49-88)	
	PAF, %	0	1.0 (0.8-1.3)	0	1.0 (0.7-1.3)	0	0.9 (0.6-1.2)	0	0.7 (0.5-0.9)	

Cancer type	Burden	20-44 y		45-54 y		55-64 y		65+ y		Overall
		Men	Women	Men	Women	Men	Women	Men	Women	
**Gallbladder**										
Age	N	2 (1-3)		12 (9-14)		24 (21-28)		68 (59-78)		106 (96-117)
	PAF, %	3.6 (3.1-4.1)		3.6 (3.2-4.0)		3.4 (3.0-3.8)		2.6 (2.3-2.9)		2.8 (2.6-3.1)
Race/ethnicity										
White	N	0 (0-0)	1 (0-2)	1 (1-3)	4 (2-5)	4 (3-5)	9 (7-12)	14 (10-18)	32 (25-41)	65 (56-76)
	PAF, %	3.1 (2.4-4.0)	3.7 (2.8-4.7)	3.0 (2.3-3.8)	3.7 (2.8-4.6)	3.0 (2.3-3.8)	3.5 (2.7-4.3)	2.3 (1.8-2.9)	2.6 (2.1-3.3)	2.7 (2.4-3.1)
Black	N	0 (0-0)	0 (0-0)	0 (0-1)	2 (1-3)	1 (1-2)	4 (3-5)	3 (2-4)	7 (5-10)	18 (15-21)
	PAF, %	4.1 (3.0-5.1)	4.1 (3.1-5.1)	3.4 (2.6-4.3)	4.1 (3.2-5.2)	3.4 (2.5-4.4)	3.9 (2.9-5.0)	2.5 (2.0-3.2)	2.8 (2.2-3.5)	3.1 (2.8-3.5)
Hispanic	N	0 (0-0)	0 (0-1)	0 (0-1)	3 (2-4)	1 (0-1)	4 (2-5)	3 (1-4)	6 (4-9)	17 (14-20)
	PAF, %	3.5 (2.7-4.4)	4.2 (3.2-5.4)	3.0 (2.3-3.7)	3.7 (2.8-4.7)	3.0 (2.3-3.7)	3.6 (2.8-4.6)	2.4 (1.9-3.0)	2.6 (2.1-3.2)	2.9 (2.6-3.3)
Other	N	0 (0-0)	0 (0-0)	0 (0-0)	1 (0-1)	0 (0-0)	1 (1-1)	1 (1-2)	3 (1-4)	6 (5-8)
	PAF, %	3.1 (2.3-3.9)	3.5 (2.7-4.4)	2.7 (2.0-3.5)	3.3 (2.5-4.2)	2.8 (2.0-3.5)	3.0 (2.3-3.8)	2.1 (1.6-2.6)	2.6 (2.1-3.3)	2.7 (2.3-3.0)
Total	N	1 (1-1)	1 (0-2)	3 (2-4)	9 (7-11)	7 (5-8)	18 (14-21)	20 (16-25)	47 (39-57)	
	PAF, %	3.3 (2.8-3.9)	3.9 (3.2-4.5)	3.0 (2.6-3.6)	3.8 (3.3-4.3)	3.1 (2.6-3.6)	3.5 (3.0-4.1)	2.4 (2.0-2.8)	2.7 (2.3-3.1)	
**All Cancers**										
Age	N	4885 (4544-5244)		12143 (11307-12959)		21147 (19670-22554)		41874 (38684-44959)		80110 (76316-83657)
	PAF, %	4.3 (4.0-4.6)		6.3 (5.9-6.7)		5.5 (5.1-5.8)		5.0 (4.6-5.3)		5.2 (5.0-5.5)
Race/ethnicity										
White	N	1494 (1260-1719)	1575 (1355-1792)	4510 (3799-5159)	3836 (3380-4279)	8527 (7217-9614)	6719 (5975-7418)	16246 (13974-18552)	16847 (14538-18787)	59732 (55985-63145)
	PAF, %[b]	5.6 (4.7-6.4)	3.3 (2.8-3.7)	7.8 (6.6-9.0)	4.8 (4.3-5.4)	5.6 (4.8-6.4)	4.8 (4.3-5.3)	4.5 (3.8-5.1)	5.2 (4.5-5.8)	5.0 (4.7-5.3)
Black	N	323 (266-393)	366 (301-441)	879 (760-1002)	933 (822-1049)	1633 (1411-1839)	1531 (1357-1687)	2037 (1750-2329)	2262 (1955-2568)	9955 (9434-10500)
	PAF, %[b]	7.2 (5.8-8.9)	4.0 (3.3-4.8)	7.5 (6.5-8.6)	6.4 (5.6-7.3)	5.7 (4.9-6.5)	6.5 (5.8-7.2)	5.3 (4.5-6.1)	6.1 (5.3-7.0)	5.9 (5.6-6.3)
Hispanic	N	352 (290-424)	435 (366-514)	653 (553-757)	684 (590-774)	969 (820-1106)	864 (758-967)	1503 (1268-1725)	1410 (1207-1620)	6874 (6472-7236)
	PAF, %[b]	5.8 (4.7-7.1)	3.9 (3.2-4.6)	8.5 (7.2-10.0)	5.4 (4.7-6.2)	6.9 (5.9-7.9)	5.9 (5.1-6.6)	6.0 (5.0-6.9)	5.9 (5.0-6.8)	6.0 (5.6-6.3)
Other	N	159 (122-198)	184 (140-232)	319 (261-380)	345 (295-401)	479 (402-560)	451 (383-521)	757 (626-917)	820 (680-971)	3519 (3298-3794)
	PAF, %[b]	7.5 (5.6-9.7)	3.5 (2.7-4.6)	10.1 (8.1-12.2)	5.4 (4.6-6.4)	7.5 (6.3-8.9)	5.6 (4.7-6.5)	5.4 (4.4-6.6)	6.1 (5.0-7.3)	6.0 (5.5-6.5)
Total	N	2323 (2063-2587)	2559 (2311-2803)	6359 (5664-6999)	5810 (5318-6286)	11614 (10228-12768)	9563 (8789-10295)	20546 (18159-22903)	21316 (19042-23332)	
	PAF, %[b]	5.9 (5.2-6.6)	3.5 (3.1-3.8)	8.0 (7.1-8.8)	5.2 (4.7-5.6)	5.8 (5.1-6.4)	5.2 (4.7-5.5)	4.7 (4.1-5.2)	5.3 (4.8-5.9)	

PAF: Population Attributable Fraction; UI: Uncertainty Intervals

Table 6. Estimated Number of New Cancer Cases Associated with Suboptimal Dietary Intake Among US Adults in 2015, by Dietary Factor and Population Subgroups

Dietary Factor	Burden*	20-44 y		45-54 y		55-64 y		65+ y		Overall
		Men	Women	Men	Women	Men	Women	Men	Women	
**Whole grains (<125 g/d)**										
Overall by age	N	1793 (1516-2077)		4202 (3518-4798)		6545 (5525-7596)		15268 (12517-17761)		27763 (24734-30596)
	PAF %	1.6 (1.3-1.8)		2.2 (1.8-2.5)		1.7 (1.4-2.0)		1.8 (1.5-2.1)		1.8 (1.6-2.0)
Race/ethnicity										
White	N	592 (396-779)	554 (359-735)	1561 (1033-1993)	1265 (870-1599)	2588 (1817-3338)	1985 (1384-2522)	5995 (4040-7847)	6032 (4134-7639)	20531 (17437-23202)
	PAF %	2.2 (1.5-2.9)	1.2 (0.8-1.5)	2.7 (1.8-3.5)	1.6 (1.1-2.0)	1.7 (1.2-2.2)	1.4 (1.0-1.8)	1.6 (1.1-2.2)	1.9 (1.3-2.4)	1.7 (1.5-2.0)
Black	N	122 (80-165)	128 (84-175)	323 (221-418)	323 (218-421)	542 (363-709)	482 (324-626)	729 (506-973)	800 (564-1031)	3437 (3038-3865)
	PAF %	2.7 (1.8-3.7)	1.4 (0.9-1.9)	2.8 (1.9-3.6)	2.2 (1.5-2.9)	1.9 (1.3-2.5)	2.0 (1.4-2.6)	1.9 (1.3-2.5)	2.2 (1.5-2.8)	2.1 (1.8-2.3)
Hispanic	N	147 (96-197)	147 (96-193)	260 (182-343)	245 (168-319)	368 (246-479)	285 (201-370)	610 (411-806)	533 (361-695)	2582 (2268-2903)
	PAF %	2.4 (1.6-3.3)	1.3 (0.8-1.7)	3.4 (2.4-4.5)	1.9 (1.3-2.5)	2.6 (1.7-3.4)	1.9 (1.4-2.5)	2.4 (1.6-3.2)	2.2 (1.5-2.9)	2.2 (2.0-2.5)
Other	N	52 (33-74)	59 (36-89)	115 (76-155)	112 (76-151)	174 (116-234)	138 (93-182)	287 (193-397)	268 (182-375)	1210 (1048-1373)
	PAF %	2.4 (1.5-3.6)	1.1 (0.7-1.7)	3.6 (2.4-5.0)	1.8 (1.2-2.4)	2.7 (1.8-3.7)	1.7 (1.1-2.3)	2.0 (1.4-2.8)	2.0 (1.3-2.8)	2.1 (1.8-2.3)
Total	N	916 (700-1108)	884 (693-1084)	2258 (1707-2700)	1945 (1520-2319)	3664 (2859-4480)	2888 (2259-3469)	7625 (5628-9512)	7646 (5673-9273)	
	PAF %	2.3 (1.8-2.8)	1.2 (0.9-1.5)	2.8 (2.1-3.4)	1.7 (1.3-2.1)	1.8 (1.4-2.2)	1.6 (1.2-1.9)	1.7 (1.3-2.2)	1.9 (1.4-2.3)	
**Total dairy products (<3 serving/d)**										
Overall by age	N	1074 (926-1209)		2622 (2257-2944)		4104 (3555-4697)		10161 (8642-11764)		17962 (16317-19572)
	PAF %	0.9 (0.8-1.1)		1.4 (1.2-1.5)		1.1 (0.9-1.2)		1.2 (1.0-1.4)		1.2 (1.1-1.3)
Race/ethnicity										
White	N	346 (249-443)	318 (225-408)	949 (689-1224)	746 (547-950)	1590 (1136-2051)	1170 (826-1474)	3854 (2815-4905)	4022 (2900-5097)	12950 (11309-14587)
	PAF %	1.3 (0.9-1.7)	0.7 (0.5-0.8)	1.7 (1.2-2.1)	0.9 (0.7-1.2)	1.1 (0.7-1.4)	0.8 (0.6-1.1)	1.1 (0.8-1.4)	1.2 (0.9-1.6)	1.1 (1.0-1.2)
Black	N	82 (57-111)	88 (62-119)	230 (168-300)	227 (167-292)	390 (282-490)	346 (247-428)	579 (417-747)	607 (441-779)	2554 (2249-2829)
	PAF %	1.8 (1.2-2.5)	1.0 (0.7-1.3)	2.0 (1.4-2.6)	1.6 (1.2-2.0)	1.4 (1.0-1.7)	1.5 (1.1-1.8)	1.5 (1.1-1.9)	1.6 (1.2-2.1)	1.5 (1.3-1.7)
Hispanic	N	82 (57-111)	75 (53-103)	152 (107-200)	143 (104-185)	221 (159-281)	168 (118-216)	361 (256-469)	332 (235-431)	1536 (1372-1715)
	PAF %	1.3 (0.9-1.8)	0.7 (0.5-0.9)	2.0 (1.4-2.6)	1.1 (0.8-1.5)	1.6 (1.1-2.0)	1.1 (0.8-1.5)	1.4 (1.0-1.9)	1.4 (1.0-1.8)	1.3 (1.2-1.5)
Other	N	37 (23-52)	41 (25-60)	87 (60-117)	81 (57-107)	135 (94-177)	103 (72-138)	224 (154-301)	219 (149-294)	929 (807-1063)
	PAF %	1.7 (1.1-2.5)	0.8 (0.5-1.2)	2.7 (1.9-3.7)	1.3 (0.9-1.7)	2.1 (1.4-2.8)	1.3 (0.9-1.7)	1.6 (1.1-2.2)	1.6 (1.1-2.2)	1.6 (1.4-1.8)
Total	N	548 (440-654)	522 (425-623)	1422 (1134-1704)	1202 (984-1420)	2330 (1875-2806)	1789 (1430-2128)	5004 (3904-6115)	5174 (4070-6314)	
	PAF %	1.4 (1.1-1.7)	0.7 (0.6-0.8)	1.8 (1.4-2.1)	1.1 (0.9-1.3)	1.2 (0.9-1.4)	1.0 (0.8-1.2)	1.1 (0.9-1.4)	1.3 (1.0-1.6)	
**High processed meats (>0 g/d)**										
Overall by age	N	863 (693-1052)		1999 (1608-2365)		3438 (2768-4127)		8219 (6394-10259)		14524 (12473-16752)
	PAF %	0.8 (0.6-0.9)		1.0 (0.8-1.2)		0.9 (0.7-1.1)		1.0 (0.8-1.2)		0.9 (0.8-1.1)
Race/ethnicity										
White	N	223 (121-331)	319 (192-453)	572 (319-825)	748 (457-1042)	1089 (608-1572)	1289 (835-1759)	2525 (1487-3626)	3882 (2238-5444)	10650 (8626-12897)
	PAF %	0.8 (0.5-1.2)	0.7 (0.4-0.9)	1.0 (0.6-1.4)	0.9 (0.6-1.3)	0.7 (0.4-1.0)	0.9 (0.6-1.3)	0.7 (0.4-1.0)	1.2 (0.7-1.7)	0.9 (0.7-1.1)
Black	N	50 (28-75)	77 (46-113)	137 (76-195)	212 (131-292)	251 (147-354)	316 (207-429)	371 (214-530)	546 (315-753)	1964 (1623-2284)
	PAF %	1.1 (0.6-1.7)	0.8 (0.5-1.2)	1.2 (0.7-1.7)	1.5 (0.9-2.0)	0.9 (0.5-1.2)	1.3 (0.9-1.8)	1.0 (0.6-1.4)	1.5 (0.8-2.0)	1.2 (1.0-1.4)
Hispanic	N	54 (31-79)	81 (50-116)	95 (55-135)	127 (76-175)	146 (85-204)	164 (101-222)	251 (147-357)	316 (186-435)	1235 (1028-1415)
	PAF %	0.9 (0.5-1.3)	0.7 (0.4-1.0)	1.2 (0.7-1.8)	1.0 (0.6-1.4)	1.0 (0.6-1.5)	1.1 (0.7-1.5)	1.0 (0.6-1.4)	1.3 (0.8-1.8)	1.1 (0.9-1.2)
Other	N	20 (11-32)	37 (22-56)	41 (23-62)	65 (40-96)	81 (49-117)	97 (61-133)	136 (80-200)	205 (125-293)	686 (567-811)
	PAF %	1.0 (0.5-1.5)	0.7 (0.4-1.1)	1.3 (0.7-1.9)	1.0 (0.6-1.5)	1.3 (0.8-1.8)	1.2 (0.8-1.7)	1.0 (0.6-1.4)	1.5 (0.9-2.2)	1.2 (1.0-1.4)
Total	N	349 (234-459)	516 (374-657)	845 (575-1110)	1149 (834-1441)	1567 (1053-2068)	1870 (1402-2333)	3291 (2231-4392)	4943 (3256-6587)	

	PAF %	0.9 (0.6-1.2)	0.7 (0.5-0.9)	1.1 (0.7-1.4)	1.0 (0.7-1.3)	0.8 (0.5-1.0)	1.0 (0.8-1.3)	0.7 (0.5-1.0)	1.2 (0.8-1.6)	
**Insufficient vegetables (<400 g/d)**										
Overall by age	N	664 (542-783)		2003 (1547-2412)		4154 (3233-4937)		5838 (4539-7056)		12663 (11026-14119)
	PAF %	0.6 (0.5-0.7)		1.0 (0.8-1.3)		1.1 (0.8-1.3)		0.7 (0.5-0.8)		0.8 (0.7-0.9)
Race/ethnicity										
White	N	236 (149-336)	185 (121-249)	1027 (608-1391)	487 (345-629)	2305 (1459-3078)	982 (695-1249)	3102 (1900-4169)	1744 (1213-2266)	10067 (8464-11509)
	PAF %	0.9 (0.6-1.2)	0.4 (0.3-0.5)	1.8 (1.1-2.4)	0.6 (0.4-0.8)	1.5 (1.0-2.0)	0.7 (0.5-0.9)	0.9 (0.5-1.1)	0.5 (0.4-0.7)	0.8 (0.7-1.0)
Black	N	48 (26-72)	37 (23-55)	146 (93-196)	89 (63-116)	319 (216-420)	175 (128-218)	301 (205-389)	182 (135-232)	1296 (1125-1463)
	PAF %	1.1 (0.6-1.6)	0.4 (0.3-0.6)	1.3 (0.8-1.7)	0.6 (0.4-0.8)	1.1 (0.7-1.5)	0.7 (0.5-0.9)	0.8 (0.5-1.0)	0.5 (0.4-0.6)	0.8 (0.7-0.9)
Hispanic	N	43 (26-65)	49 (30-69)	96 (62-130)	59 (43-76)	157 (104-206)	84 (62-109)	212 (143-286)	123 (86-164)	824 (716-932)
	PAF %	0.7 (0.4-1.1)	0.4 (0.3-0.6)	1.3 (0.8-1.7)	0.5 (0.3-0.6)	1.1 (0.8-1.5)	0.6 (0.4-0.7)	0.8 (0.6-1.1)	0.5 (0.4-0.7)	0.7 (0.6-0.8)
Other	N	36 (19-55)	24 (14-37)	58 (34-84)	42 (29-57)	71 (42-105)	60 (44-79)	101 (64-140)	79 (53-107)	471 (406-551)
	PAF %	1.7 (0.9-2.7)	0.5 (0.3-0.7)	1.8 (1.1-2.6)	0.7 (0.5-0.9)	1.1 (0.6-1.6)	0.7 (0.5-1.0)	0.7 (0.4-1.0)	0.6 (0.4-0.8)	0.8 (0.7-0.9)
Total	N	365 (274-470)	295 (228-371)	1330 (912-1692)	676 (526-816)	2850 (2007-3624)	1305 (1005-1571)	3718 (2460-4790)	2131 (1576-2643)	
	PAF %	0.9 (0.7-1.2)	0.4 (0.3-0.5)	1.7 (1.1-2.1)	0.6 (0.5-0.7)	1.4 (1.0-1.8)	0.7 (0.5-0.8)	0.8 (0.6-1.1)	0.5 (0.4-0.7)	
**Insufficient fruits (<300 g/d)**										
Overall by age	N	463 (346-577)		1335 (950-1721)		2682 (1956-3388)		3414 (2498-4360)		7927 (6752-9146)
	PAF %	0.4 (0.3-0.5)		0.7 (0.5-0.9)		0.7 (0.5-0.9)		0.4 (0.3-0.5)		0.5 (0.4-0.6)
Race/ethnicity										
White	N	141 (59-226)	149 (89-210)	581 (208-947)	403 (272-531)	1245 (512-1884)	850 (598-1109)	1532 (676-2362)	1259 (842-1719)	6143 (4997-7456)
	PAF %	0.5 (0.2-0.8)	0.3 (0.2-0.4)	1.0 (0.4-1.6)	0.5 (0.3-0.7)	0.8 (0.3-1.2)	0.6 (0.4-0.8)	0.4 (0.2-0.6)	0.4 (0.3-0.5)	0.5 (0.4-0.6)
Black	N	30 (13-51)	30 (17-45)	85 (42-128)	75 (52-101)	185 (95-271)	156 (113-200)	171 (94-253)	148 (111-187)	881 (736-1024)
	PAF %	0.7 (0.3-1.1)	0.3 (0.2-0.5)	0.7 (0.4-1.1)	0.5 (0.4-0.7)	0.6 (0.3-0.9)	0.7 (0.5-0.8)	0.4 (0.2-0.7)	0.4 (0.3-0.5)	0.5 (0.4-0.6)
Hispanic	N	29 (14-47)	43 (28-62)	59 (28-89)	62 (46-80)	96 (51-144)	85 (63-108)	118 (62-174)	101 (72-135)	594 (503-686)
	PAF %	0.5 (0.2-0.8)	0.4 (0.2-0.6)	0.8 (0.4-1.2)	0.5 (0.4-0.6)	0.7 (0.4-1.0)	0.6 (0.4-0.7)	0.5 (0.2-0.7)	0.4 (0.3-0.6)	0.5 (0.4-0.6)
Other	N	20 (6-36)	18 (10-28)	31 (13-53)	35 (23-47)	41 (20-64)	43 (29-58)	49 (25-77)	54 (37-75)	294 (244-350)
	PAF %	1.0 (0.3-1.7)	0.3 (0.2-0.5)	1.0 (0.4-1.7)	0.5 (0.4-0.7)	0.6 (0.3-1.0)	0.5 (0.4-0.7)	0.4 (0.2-0.6)	0.4 (0.3-0.6)	0.5 (0.4-0.6)
Total	N	223 (131-316)	241 (176-303)	758 (381-1129)	575 (440-697)	1557 (835-2226)	1135 (862-1388)	1866 (1029-2705)	1560 (1138-2013)	
	PAF %	0.6 (0.3-0.8)	0.3 (0.2-0.4)	0.9 (0.5-1.4)	0.5 (0.4-0.6)	0.8 (0.4-1.1)	0.6 (0.5-0.8)	0.4 (0.2-0.6)	0.4 (0.3-0.5)	
**High red meats (>14.3 g/d)**										
Overall by age	N	359 (233-498)		856 (523-1172)		1410 (942-1940)		3071 (1675-4610)		5689 (4168-7332)
	PAF %	0.3 (0.2-0.4)		0.4 (0.3-0.6)		0.4 (0.2-0.5)		0.4 (0.2-0.5)		0.4 (0.3-0.5)
Race/ethnicity										
White	N	83 (14-154)	140 (29-236)	223 (34-416)	350 (101-587)	407 (84-715)	590 (225-949)	897 (125-1648)	1582 (381-2817)	4250 (2815-5943)
	PAF %	0.3 (0.1-0.6)	0.3 (0.1-0.5)	0.4 (0.1-0.7)	0.4 (0.1-0.7)	0.3 (0.1-0.5)	0.4 (0.2-0.7)	0.2 (0.0-0.5)	0.5 (0.1-0.9)	0.4 (0.2-0.5)
Black	N	17 (3-31)	30 (6-55)	43 (5-80)	76 (21-135)	78 (15-143)	124 (40-203)	98 (17-188)	178 (43-314)	649 (456-844)
	PAF %	0.4 (0.1-0.7)	0.3 (0.1-0.6)	0.4 (0.0-0.7)	0.5 (0.1-0.9)	0.3 (0.1-0.5)	0.5 (0.2-0.9)	0.3 (0.0-0.5)	0.5 (0.1-0.8)	0.4 (0.3-0.5)
Hispanic	N	24 (3-47)	42 (14-75)	44 (7-79)	72 (22-116)	63 (13-111)	85 (31-146)	92 (14-170)	130 (34-224)	552 (390-730)
	PAF %	0.4 (0.1-0.8)	0.4 (0.1-0.7)	0.6 (0.1-1.1)	0.6 (0.2-0.9)	0.4 (0.1-0.8)	0.6 (0.2-1.0)	0.4 (0.1-0.7)	0.5 (0.1-0.9)	0.5 (0.3-0.6)
Other	N	7 (1-14)	14 (4-26)	16 (2-32)	33 (10-57)	23 (4-42)	40 (15-67)	40 (6-78)	66 (16-116)	240 (168-312)
	PAF %	0.3 (0.1-0.7)	0.3 (0.1-0.5)	0.5 (0.1-1.0)	0.5 (0.2-0.9)	0.4 (0.1-0.7)	0.5 (0.2-0.8)	0.3 (0.0-0.6)	0.5 (0.1-0.9)	0.4 (0.3-0.5)
Total	N	131 (58-207)	226 (117-329)	326 (138-516)	530 (283-777)	571 (243-905)	842 (471-1224)	1129 (362-1883)	1965 (765-3223)	
	PAF %	0.3 (0.1-0.5)	0.3 (0.2-0.4)	0.4 (0.2-0.6)	0.5 (0.3-0.7)	0.3 (0.1-0.5)	0.5 (0.3-0.7)	0.3 (0.1-0.4)	0.5 (0.2-0.8)	
**High sugar-sweetened beverages (>0 serving/d)**										
Overall by age	N	268 (231-305)		617 (536-700)		1137 (985-1305)		1092 (961-1260)		3119 (2891-3352)
	PAF %	0.2 (0.2-0.3)		0.3 (0.3-0.4)		0.3 (0.3-0.3)		0.1 (0.1-0.1)		0.2 (0.2-0.2)
Race/ethnicity										

White	N	39 (28-54)	111 (82-143)	102 (74-137)	316 (243-389)	211 (159-271)	580 (443-731)	232 (175-301)	579 (458-719)	2178 (1959-2414)
	PAF %	0.1 (0.1-0.2)	0.2 (0.2-0.3)	0.2 (0.1-0.2)	0.4 (0.3-0.5)	0.1 (0.1-0.2)	0.4 (0.3-0.5)	0.1 (0.0-0.1)	0.2 (0.1-0.2)	0.2 (0.2-0.2)
Black	N	14 (9-19)	25 (18-32)	25 (18-34)	63 (49-81)	62 (45-82)	135 (104-167)	38 (27-50)	108 (87-134)	472 (420-521)
	PAF %	0.3 (0.2-0.4)	0.3 (0.2-0.4)	0.2 (0.2-0.3)	0.4 (0.3-0.6)	0.2 (0.2-0.3)	0.6 (0.4-0.7)	0.1 (0.1-0.1)	0.3 (0.2-0.4)	0.3 (0.3-0.3)
Hispanic	N	14 (9-19)	48 (35-62)	21 (15-29)	60 (46-75)	30 (21-41)	86 (66-106)	30 (22-40)	59 (48-72)	349 (316-383)
	PAF %	0.2 (0.2-0.3)	0.4 (0.3-0.6)	0.3 (0.2-0.4)	0.5 (0.4-0.6)	0.2 (0.2-0.3)	0.6 (0.5-0.7)	0.1 (0.1-0.2)	0.2 (0.2-0.3)	0.3 (0.3-0.3)
Other	N	4 (2-6)	12 (8-16)	7 (5-10)	20 (14-25)	11 (8-16)	22 (16-28)	10 (7-14)	32 (25-41)	119 (105-132)
	PAF %	0.2 (0.1-0.3)	0.2 (0.2-0.3)	0.2 (0.1-0.3)	0.3 (0.2-0.4)	0.2 (0.1-0.3)	0.3 (0.2-0.4)	0.1 (0.1-0.1)	0.2 (0.2-0.3)	0.2 (0.2-0.2)
Total	N	71 (57-88)	196 (162-230)	155 (126-197)	460 (388-538)	314 (256-379)	822 (684-978)	311 (250-382)	778 (656-920)	
	PAF %	0.2 (0.1-0.2)	0.3 (0.2-0.3)	0.2 (0.2-0.2)	0.4 (0.3-0.5)	0.2 (0.1-0.2)	0.4 (0.4-0.5)	0.1 (0.1-0.1)	0.2 (0.2-0.2)	

Supplementary Figures

Figure 1. Proportion of New Cancer Cases (%) Associated with Suboptimal Diet among U.S. Adults in 2015 by Cancer Type

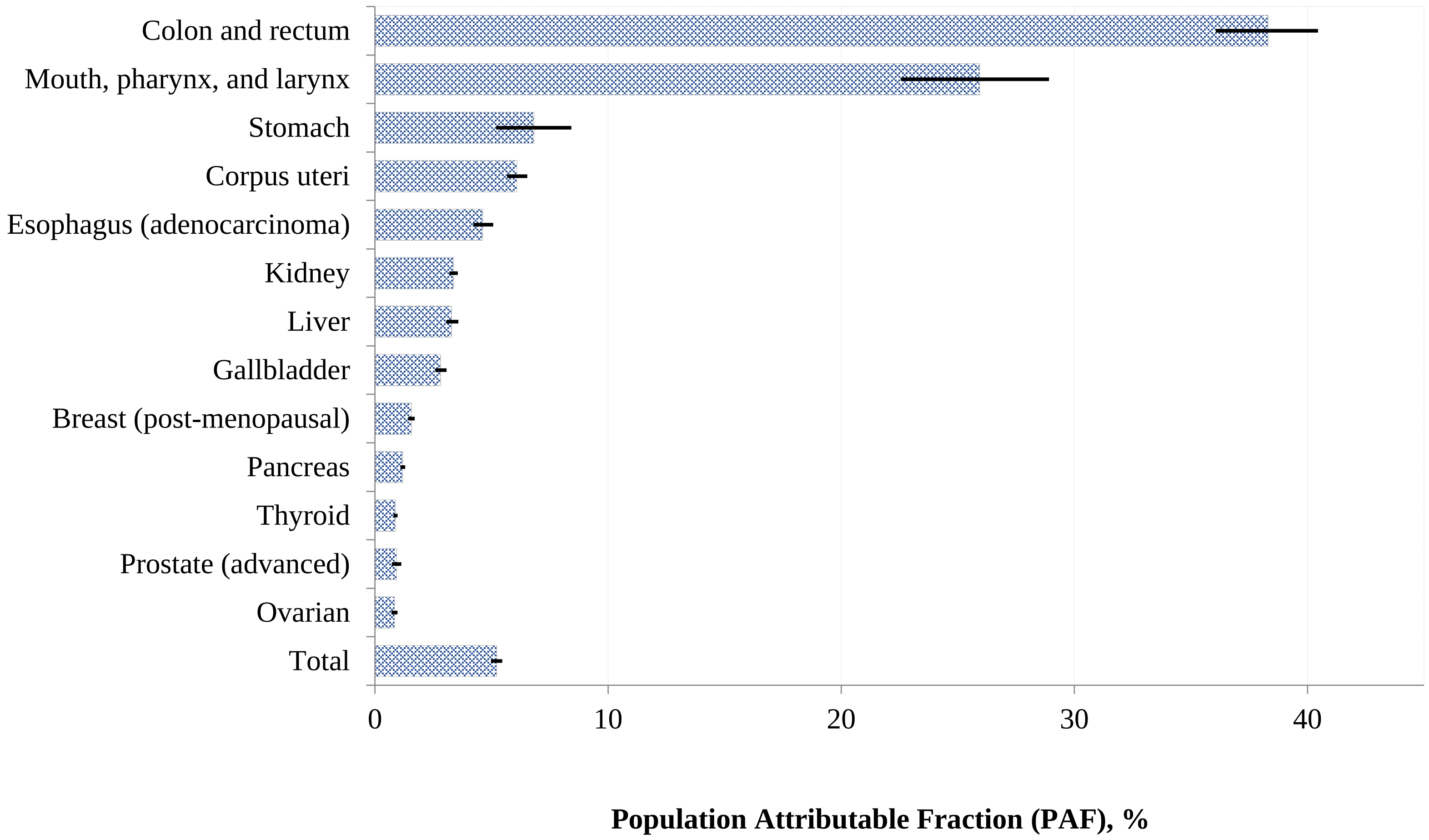

Figure 2. Proportion of New Cancer Cases (%) Associated with Suboptimal Diet Among U.S. Adults in 2015, by Dietary Factors

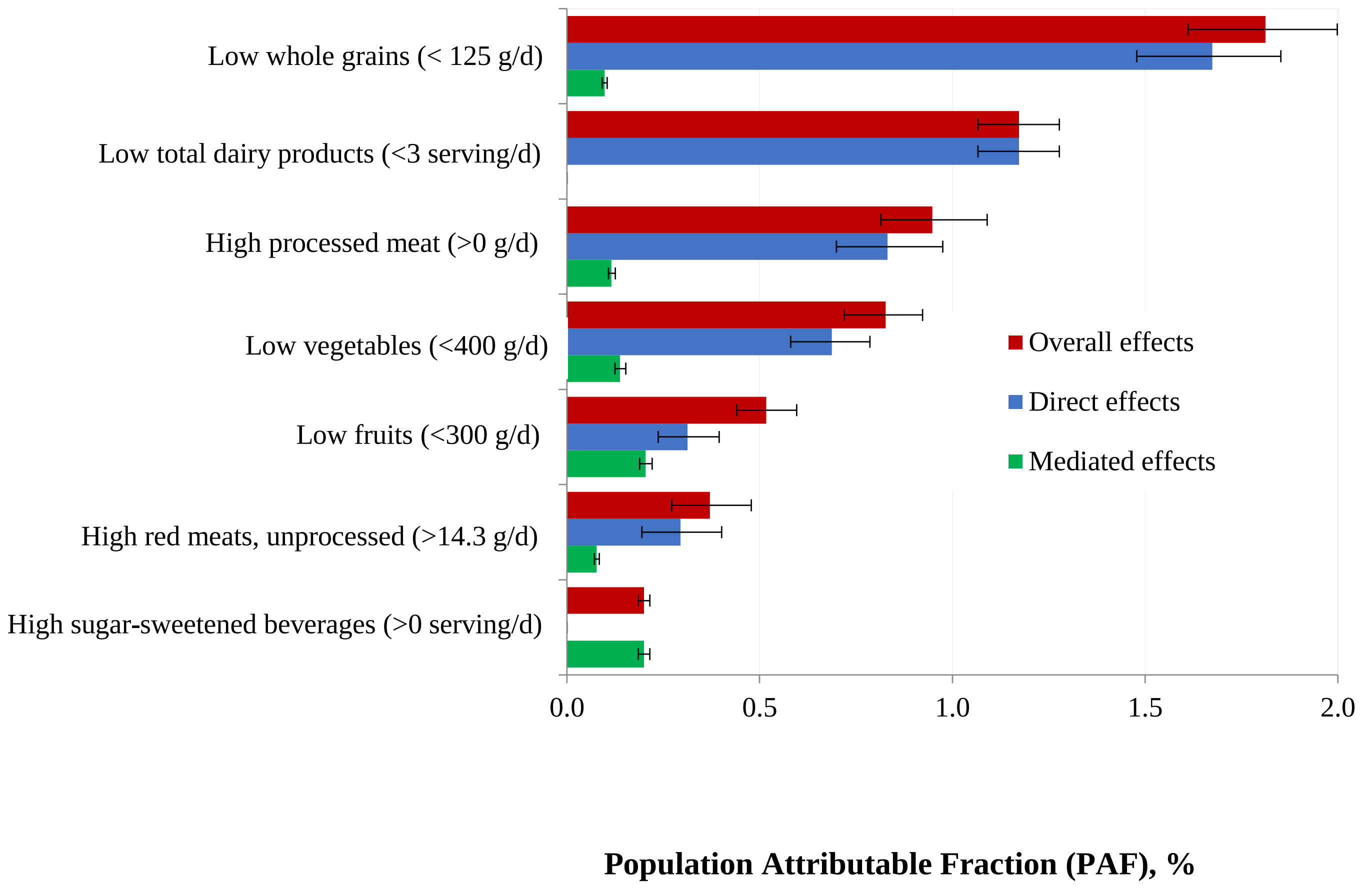

Supplementary References

1. Vander Hoorn S, Ezzati M, Rodgers A, Lopez AD, Murray CJ. Estimating attributable burden of disease from exposure and hazard data. In: Murray CJ, Ezzati M, Lopez AD, Rodgers A, Vander Hoorn S, eds. *Comparative Quantication of Health Risks: Global and Regional Burden of Disease Attributable to Selected Major Risk Factors.* Vol 2. 2003/06/05 ed. Geneva: World Health Organization; 2004:2129-2140.
2. Murray CJ, Ezzati M, Flaxman AD, et al. GBD 2010: a multi-investigator collaboration for global comparative descriptive epidemiology. *Lancet.* 2012;380(9859):2055-2058.
3. Tooze JA, Kipnis V, Buckman DW, et al. A mixed-effects model approach for estimating the distribution of usual intake of nutrients: the NCI method. *Statistics in medicine.* 2010;29(27):2857-2868.
4. Herrick KA, Rossen LM, Parsons R, Dodd KW. Estimating Usual Dietary In take From National Health and Nut rition Examination Survey Data Using the National Cancer Institute Method. *Vital and health statistics Series 2, Data evaluation and methods research.* 2018;178(2):1-63.
5. Tooze JA, Midthune D, Dodd KW, et al. A new statistical method for estimating the usual intake of episodically consumed foods with application to their distribution. *Journal of the American Dietetic Association.* 2006;106(10):1575-1587.
6. Lim SS, Vos T, Flaxman AD, et al. A comparative risk assessment of burden of disease and injury attributable to 67 risk factors and risk factor clusters in 21 regions, 1990-2010: a systematic analysis for the Global Burden of Disease Study 2010. *Lancet.* 2012;380(9859):2224-2260.
7. Micha R, Penalvo JL, Cudhea F, Imamura F, Rehm CD, Mozaffarian D. Association Between Dietary Factors and Mortality From Heart Disease, Stroke, and Type 2 Diabetes in the United States. *Jama.* 2017;317(9):912-924.
8. Mozaffarian D, Fahimi S, Singh GM, et al. Global sodium consumption and death from cardiovascular causes. *The New England journal of medicine.* 2014;371(7):624-634.
9. Mozaffarian D, Hao T, Rimm EB, Willett WC, Hu FB. Changes in diet and lifestyle and long-term weight gain in women and men. *The New England journal of medicine.* 2011;364(25):2392-2404.
10. Lauby-Secretan B, Scoccianti C, Loomis D, Grosse Y, Bianchini F, Straif K. Body Fatness and Cancer--Viewpoint of the IARC Working Group. *The New England journal of medicine.* 2016;375(8):794-798.
11. Navarrete-Munoz EM, Wark PA, Romaguera D, et al. Sweet-beverage consumption and risk of pancreatic cancer in the European Prospective Investigation into Cancer and Nutrition (EPIC). *The American journal of clinical nutrition.* 2016;104(3):760-768.
12. Stepien M, Duarte-Salles T, Fedirko V, et al. Consumption of soft drinks and juices and risk of liver and biliary tract cancers in a European cohort. *European journal of nutrition.* 2016;55(1):7-20.
13. World Cancer Research Fund/ American Institute for Cancer Research. Diet, Nutrition, Physical Activity, and Cancer: a Global Perspective. Contiuous Update Project Expert Report 2018. https://www.wcrf.org/dietandcancer. Accessed March 2, 2019.
14. World Cancer Research Fund/ American Institute for Cancer Research. Continuous Update Project Expert Report: Diet, Nutrition, Physical activity and Colorectal Cancer. 2018; https://www.wcrf.org/sites/default/files/Colorectal-cancer-report.pdf. Accessed March 2, 2019.
15. World Cancer Research Fund/ American Institute for Cancer Research. Continuous Update Project Expert Report: Diet, Nutrition, Physical Activity and Stomach Cancer. 2018; https://www.wcrf.org/sites/default/files/Stomach-cancer-report.pdf. Accessed March 2, 2019.
16. World Cancer Research Fund/ American Institute for Cancer Research. Continuous Update Project Expert Report: Diet, nutrition and physical activity: Energy Balance and body fatness. 2018; https://www.wcrf.org/sites/default/files/energy-balance-and-body-fatness.pdf. Accessed March 2, 2019.
17. Malik VS, Schulze MB, Hu FB. Intake of sugar-sweetened beverages and weight gain: a systematic review. *Am J Clin Nutr.* 2006;84(2):274-288.
18. Mozaffarian D, Hao T, Rimm EB, Willett WC, Hu FB. Changes in diet and lifestyle and long-term weight gain in women and men. *The New England journal of medicine.* 2011;364(25):2392-2404.

19. Te Morenga L, Mallard S, Mann J. Dietary sugars and body weight: systematic review and meta-analyses of randomised controlled trials and cohort studies. *BMJ.* 2013;346:e7492.
20. Satija A, Yu E, Willett WC, Hu FB. Understanding nutritional epidemiology and its role in policy. *Advances in nutrition (Bethesda, Md).* 2015;6(1):5-18.
21. Martinez ME, Marshall JR, Giovannucci E. Diet and cancer prevention: the roles of observation and experimentation. *Nature reviews Cancer.* 2008;8(9):694-703.
22. Hershman DL, Wright JD. Comparative effectiveness research in oncology methodology: observational data. *Journal of clinical oncology : official journal of the American Society of Clinical Oncology.* 2012;30(34):4215-4222.
23. Rosner B, Spiegelman D, Willett WC. Correction of logistic regression relative risk estimates and confidence intervals for random within-person measurement error. *Am J Epidemiol.* 1992;136(11):1400-1413.
24. Willett WC. *Nutritional Epidemiology*. Second ed. New York: Oxford University Press; 1998.
25. Research WCRFAIfC. Continuous update and systematic literature review of randomised controlled trials, prospective studies and case-control studies on food, nutrition, physical activity and cancers of mouth, pharynx and larynx. 2013. http://www.dietandcancerreport.org/cup/report_overview/index.php.
26. Wallace BC, Small K, Brodley CE, Lau J, Trikalinos TA. Deploying an interactive machine learning system in an evidence-based practice center: abstrackr. *In Proc of the ACM International Health Informatics Symposium (IHI).* 2012:819-824.
27. Orsini N, Bellocco R, Greenland S. Generalized least squares for trend estimation of summarized dose-response data *Stata J.* 2006;6:40-57.
28. Greenland S, Longnecker MP. Methods for trend estimation from summarized dose-response data, with applications to meta-analysis. *Am J Epidemiol.* 1992;135(11):1301-1309.
29. Higgins JP, Thompson SG. Quantifying heterogeneity in a meta-analysis. *Stat Med.* 2002;21:1539-1558.
30. Maasland DH, van den Brandt PA, Kremer B, Goldbohm RA, Schouten LJ. Consumption of vegetables and fruits and risk of subtypes of head-neck cancer in the Netherlands Cohort Study. *International journal of cancer.* 2015;136(5):E396-409.
31. Boeing H, Dietrich T, Hoffmann K, et al. Intake of fruits and vegetables and risk of cancer of the upper aero-digestive tract: the prospective EPIC-study. *Cancer causes & control : CCC.* 2006;17(7):957-969.
32. Freedman ND, Park Y, Subar AF, et al. Fruit and vegetable intake and head and neck cancer risk in a large United States prospective cohort study. *International journal of cancer.* 2008;122(10):2330-2336.
33. Chyou PH, Nomura AM, Stemmermann GN. Diet, alcohol, smoking and cancer of the upper aerodigestive tract: a prospective study among Hawaii Japanese men. *International journal of cancer.* 1995;60(5):616-621.
34. Surveillance Epidemiology and End Results (SEER) Program. Cancer Stat Facts: Prostate Cancer. 2007-2013; https://seer.cancer.gov/statfacts/html/prost.html. Accessed March 2, 2019.
35. Fang X, Wei J, He X, et al. Landscape of dietary factors associated with risk of gastric cancer: A systematic review and dose-response meta-analysis of prospective cohort studies. *Eur J Cancer.* 2015;51(18):2820-2832.
